# Hippo-YAP signaling controls lineage differentiation of mouse embryonic stem cells through modulating the formation of super-enhancers

**DOI:** 10.1093/nar/gkaa482

**Published:** 2020-06-08

**Authors:** Xiang Sun, Zhijun Ren, Yixian Cun, Cai Zhao, Xianglin Huang, Jiajian Zhou, Rong Hu, Xiaoxi Su, Lu Ji, Peng Li, King Lun Kingston Mak, Feng Gao, Yi Yang, He Xu, Junjun Ding, Nan Cao, Shuo Li, Wensheng Zhang, Ping Lan, Hao Sun, Jinkai Wang, Ping Yuan

**Affiliations:** Department of Medical Bioinformatics, Zhongshan School of Medicine, Sun Yat-sen University, Guangzhou 510275, China; Center for Stem Cell Biology and Tissue Engineering, Key Laboratory for Stem Cells and Tissue Engineering, Ministry of Education, Sun Yat-sen University, Guangzhou 510275, China; Department of Chemical Pathology, Li Ka Shing Institute of Health Sciences, Chinese University of Hong Kong, Hong Kong; Department of Medical Bioinformatics, Zhongshan School of Medicine, Sun Yat-sen University, Guangzhou 510275, China; Center for Stem Cell Biology and Tissue Engineering, Key Laboratory for Stem Cells and Tissue Engineering, Ministry of Education, Sun Yat-sen University, Guangzhou 510275, China; Department of Medical Bioinformatics, Zhongshan School of Medicine, Sun Yat-sen University, Guangzhou 510275, China; Center for Stem Cell Biology and Tissue Engineering, Key Laboratory for Stem Cells and Tissue Engineering, Ministry of Education, Sun Yat-sen University, Guangzhou 510275, China; Center for Stem Cell Biology and Tissue Engineering, Key Laboratory for Stem Cells and Tissue Engineering, Ministry of Education, Sun Yat-sen University, Guangzhou 510275, China; Center for Stem Cell Biology and Tissue Engineering, Key Laboratory for Stem Cells and Tissue Engineering, Ministry of Education, Sun Yat-sen University, Guangzhou 510275, China; Dermatology Hospital, Southern Medical University, Guangzhou, China; Department of Chemical Pathology, Li Ka Shing Institute of Health Sciences, Chinese University of Hong Kong, Hong Kong; Guangdong Provincial Key Laboratory of Colorectal and Pelvic Floor Disease, The Sixth Affiliated Hospital of Sun Yat-sen University, Guangzhou, 510655, China; Guangdong Institute of Gastroenterology, Guangzhou, Guangdong 510655, China; Department of Chemical Pathology, Li Ka Shing Institute of Health Sciences, Chinese University of Hong Kong, Hong Kong; Department of Chemical Pathology, Li Ka Shing Institute of Health Sciences, Chinese University of Hong Kong, Hong Kong; China Hong Kong Children's Hospital, Hong Kong SAR; Department of Chemical Pathology, Li Ka Shing Institute of Health Sciences, Chinese University of Hong Kong, Hong Kong; Scientific Research Center, The Seventh Afﬁliated Hospital, Sun Yat-sen University, Shenzhen, Guangdong 518107, China; Guangzhou Regenerative Medicine and Health Guangdong Laboratory (GRMH-GDL), Guangzhou, China; Guangdong Provincial Key Laboratory of Colorectal and Pelvic Floor Disease, The Sixth Affiliated Hospital of Sun Yat-sen University, Guangzhou, 510655, China; Guangdong Institute of Gastroenterology, Guangzhou, Guangdong 510655, China; Department of Medical Bioinformatics, Zhongshan School of Medicine, Sun Yat-sen University, Guangzhou 510275, China; Center for Stem Cell Biology and Tissue Engineering, Key Laboratory for Stem Cells and Tissue Engineering, Ministry of Education, Sun Yat-sen University, Guangzhou 510275, China; Center for Stem Cell Biology and Tissue Engineering, Key Laboratory for Stem Cells and Tissue Engineering, Ministry of Education, Sun Yat-sen University, Guangzhou 510275, China; Center for Stem Cell Biology and Tissue Engineering, Key Laboratory for Stem Cells and Tissue Engineering, Ministry of Education, Sun Yat-sen University, Guangzhou 510275, China; Department of Histology and embryology, School of Basic Medical Sciences, Guangzhou Medical University, Guangzhou, Guangdong 511436, China; Center for Stem Cell Biology and Tissue Engineering, Key Laboratory for Stem Cells and Tissue Engineering, Ministry of Education, Sun Yat-sen University, Guangzhou 510275, China; Department of Medical Bioinformatics, Zhongshan School of Medicine, Sun Yat-sen University, Guangzhou 510275, China; Center for Stem Cell Biology and Tissue Engineering, Key Laboratory for Stem Cells and Tissue Engineering, Ministry of Education, Sun Yat-sen University, Guangzhou 510275, China; Cam-Su Genomic Resource Center, Soochow University, Suzhou 215123, China; Guangdong Provincial Key Laboratory of Colorectal and Pelvic Floor Disease, The Sixth Affiliated Hospital of Sun Yat-sen University, Guangzhou, 510655, China; Department of Colorectal Surgery, The Sixth Affiliated Hospital of Sun Yat-sen University, Guangzhou, Guangdong, China; Department of Chemical Pathology, Li Ka Shing Institute of Health Sciences, Chinese University of Hong Kong, Hong Kong; Department of Medical Bioinformatics, Zhongshan School of Medicine, Sun Yat-sen University, Guangzhou 510275, China; Center for Stem Cell Biology and Tissue Engineering, Key Laboratory for Stem Cells and Tissue Engineering, Ministry of Education, Sun Yat-sen University, Guangzhou 510275, China; RNA Biomedical Institute, Sun Yat-sen Memorial Hospital, Sun Yat-sen University, Guangzhou 510120, China; Center for Precision Medicine, Sun Yat-sen University, Guangzhou 510080, China; Guangdong Provincial Key Laboratory of Colorectal and Pelvic Floor Disease, The Sixth Affiliated Hospital of Sun Yat-sen University, Guangzhou, 510655, China; Guangdong Institute of Gastroenterology, Guangzhou, Guangdong 510655, China; Department of Chemical Pathology, Li Ka Shing Institute of Health Sciences, Chinese University of Hong Kong, Hong Kong

## Abstract

Hippo-YAP signaling pathway functions in early lineage differentiation of pluripotent stem cells, but the detailed mechanisms remain elusive. We found that knockout (KO) of Mst1 and Mst2, two key components of the Hippo signaling in mouse embryonic stem cells (ESCs), resulted in a disruption of differentiation into mesendoderm lineage. To further uncover the underlying regulatory mechanisms, we performed a series of ChIP-seq experiments with antibodies against YAP, ESC master transcription factors and some characterized histone modification markers as well as RNA-seq assays using wild type and Mst KO samples at ES and day 4 embryoid body stage respectively. We demonstrate that YAP is preferentially co-localized with super-enhancer (SE) markers such as Nanog, Sox2, Oct4 and H3K27ac in ESCs. The hyper-activation of nuclear YAP in Mst KO ESCs facilitates the binding of Nanog, Sox2 and Oct4 as well as H3K27ac modification at the loci where YAP binds. Moreover, Mst depletion results in novel SE formation and enhanced liquid-liquid phase-separated Med1 condensates on lineage associated genes, leading to the upregulation of these genes and the distortion of ESC differentiation. Our study reveals a novel mechanism on how Hippo-YAP signaling pathway dictates ESC lineage differentiation.

## INTRODUCTION

Pluripotent stem cells (PSCs), such as embryonic stem cells (ESCs) and induced pluripotent stem cells (iPSCs) may serve as a powerful resource for regenerative medicine, due to their characteristics of pluripotency and self-renewal. However, most clinical PSC applications remain at the trial stage, mostly because it is inefficient and expensive to obtain specific cell types for cell replacement therapy based on current knowledge and technologies on the lineage-specific differentiation of PSCs. To push PSCs toward clinic application, it is fundamentally important to unveil the detailed mechanisms on how PSCs differentiate into specific lineage cells.

The Hippo pathway is highly conserved in metazoa. A number of studies have revealed that it controls organ size by restraining cell proliferation and promoting apoptosis. It is also involved in the self-renewal and differentiation of stem cells, including ESCs (1). However, the detailed mechanisms on how this pathway controls mouse ESC differentiation has not been thoroughly studied yet.

In mammals, Hippo signaling pathway is comprised of a core kinase cascade including Mst1/Mst2 and Lats1/Lats2. Growth factors, mechanical stimuluses and cell morphology changes can activate Hippo signaling and lead to phosphorylation of Mst1/Mst2. Phosphorylated Mst1/Mst2 then activates Lats1/Lats2 by phosphorylating them, which in turn phosphorylate YAP. Phosphorylated YAP is anchored by 14–3–3 in the cytoplasm and degraded by the proteasome (2). Overall, the Hippo pathway plays a repressive role on YAP.

As a transcription co-factor, YAP usually partners with transcription factors such as TEA domain-containing (TEAD) proteins to regulate the expression of target genes. Accumulating evidences support both active and repressive roles of YAP in gene regulation. YAP/TAZ are found to activate target genes associated with cell proliferation, cell adhesion, cell migration and anti-apoptosis (3,4). In mouse ESCs, YAP and TEAD2 bind to the distal enhancer of Oct4 and activate its expression (5). Whereas, it has been reported that YAP can also function as a transcriptional co-repressor by recruiting NuRD complex in MCF10A cells (6). This is due to the fact that NuRD complex can recruit polycomb repressive complex 2 (PRC2) to deposit the repressive mark H3K27me3 to its target genes in mouse embryonic stem cells (ESCs) (7,8). Additionally, in human ESCs, YAP/TEADs, Smad2/3 and Oct4 (simplified as TSO) form a complex together with the NuRD repressive complex to suppress mesendoderm lineage genes and buffer pluripotent genes (9). Ectopic expression of YAP leads to its enhanced nuclear accumulation and disturbance of ESC differentiation (1). This is consistent with our observation that Mst KO mouse ESCs show upregulation of YAP and a preferential differentiation into neuroectoderm, but a disturbed differentiation into mesoderm and endoderm as well as their downstream lineage cells (10). Despite of this observation, the mechanism on how Hippo/YAP pathway regulates mouse ESC lineage differentiation remains unclear.

In recent years, super-enhancers (SEs) have been reported to prominently regulate genes that control cell identity (11). SE differs from typical-enhancer (TE) by its large size, extensively marked active epigenetic modification, super high binding of regulatory factors and sensitivity of perturbation. Bound by very high levels of mediators, master transcription factors, chromatin regulators and transcriptional machinery, SEs drive robust expression of cell identity related genes. The binding factors at SEs condense into membrane-less organelles, resulting in phase separation from the nucleoplasm, which can be visualized as discrete puncta in nuclei by immuno-fluorescence assay with antibodies blotting the major components, such as mediator complex subunit 1 (Med1) or Brd4. This phase separated structure compartmentalizes and concentrates transcriptional machinery within restricted regions for superior transcriptional output (12).

In this study, we found that depletion of Mst1 and Mst2 in mouse ESCs suppressed mesendoderm lineage differentiation. Through ChIP-seq assays with antibodies against YAP, Nanog, Sox2, Oct4 and H3K27ac in WT and Mst KO mouse ESCs, we observed high occupancy of YAP at Nanog, Sox2, Oct4 and H3K27ac co-marked SE loci. We demonstrate that upregulation and nuclear translocation of YAP due to Mst1 and Mst2 depletion in mouse ESCs lead to the formation of novel SEs that promote the expression of genes driving ectoderm lineage differentiation and inhibiting mesendoderm lineage differentiation. Furthermore, YAP also directs the formation of Med1 marked condensates at its binding sites through liquid-liquid phase-separation. Hence, Hippo-YAP pathway plays an important role in early cell lineage specification through SE system.

## MATERIALS AND METHODS

### Isolation and characterization of mouse embryonic stem cells

Mst1 and Mst2 double knockout (Mst KO) mouse embryos were obtained by crossing *Mst1+/– Mst2–/–* female and male C57BL/6 mice. E3.5 blastocysts were collected and seeded on mitomycin C treated MEF feeder in DMEM/F12 medium supplemented with 0.4 μM MEK inhibitor PD0325901 (Stemgent), 3 μM GSK3β inhibitor CHIR99021 (Stemgent) and 1000 U/ml Leukemia inhibitory factor (LIF, Millipore) in 5% CO_2_ incubator at 37°C. The ICM outgrowths were disassociated with 0.05% Trypsin (Invitrogen) and passaged for stable ESCs. To obtain feeder free ESCs, the ESCs were grown on a 0.2% gelatin coated plate for at least two to three passages to remove feeder contamination. For embryoid body (EB) formation, wild type ESCs and Mst KO ESCs were trypsinized into single cells and 1 × 10^6^ cells were seeded at in 10 cm non-adherent dishes in EB culture medium (DMEM supplemented with 15% FBS, 0.1 mM non-essential amino acids, 0.1 mM 2-mercaptoethanol, 2 mM glutamine and 100 U/ml penicillin/streptomycin).

### Chromatin immunoprecipitation

Chromatin immunoprecipitation was performed with antibodies (Supplementary Table S1) against YAP, Nanog, Oct4, Sox2, H3K27ac, H3K4me3, H3K27me3 and H3K36me3 using chromatin extracted from Mst KO and WT ESCs respectively. Briefly, mouse ESCs were crosslinked with fresh 1% formaldehyde for 10 minutes at room temperature followed by addition of 125mM glycine and incubation for 5 min to inactivate formaldehyde. Cells were lysed and sheared by sonicator (Branson Sonifier) in ice bath to generate 200–500 bp chromatin fragments. A 50 ul aliquot was reserved as input. The rest were incubated with 100 μl of Dyna Protein G magnetic beads (Invitrogen) that had been pre-incubated with 5 μg appropriate antibody for 12 h at 4°C. After overnight rotation, beads were washed 4 times with RIPA buffer and 1 time with TE buffer for 5 min each. Protein–chromatin complexes were eluted by elution buffer containing 1% SDS at 65°C with constant shaking for 30 min and then de-crosslinked together with input samples at 65°C overnight respectively. ChIP DNA and corresponding input DNA were further digested with RNaseA and proteinase K and purified by multiple phenol:chloroform:isoamyl alcohol extractions and ethanol precipitation. The enrichment fold of ChIP DNA relative to input DNA was measured by real-time PCR. The negative control region where the protein of interest doesn’t bind was used as baseline in ChIP-qPCR. The experiments were repeated three times independently. Student's *t*-test was used to calculate the statistical significance.

### ChIP-seq library construction

5ng-10ng of ChIP DNA and Input DNA were used separately to prepare DNA library for Illumina sequencing. Firstly, DNA fragments were repaired to blunt ends by Klenow fragments enzyme and T4 DNA polymerase and a phosphate group was added to the 5′-ends of DNA fragment by T4 PNK. Next, a single ‘A’ base was added to the repaired 3′-end with Klenow (3′→5′ exo-) for adaptor ligation. Subsequently, a pair of barcoded Truseq adapters with 3′-end ‘T’ overhang was ligated to both ends of A-tailed DNA fragment with T4 DNA ligase. Eventually, the ligation products were subjected to amplification by using adaptor primers, the resultant library with size of 250–500 bp was gel purified by QIAGEN kit to remove adaptor dimers and other contamination. After quantification and quality control, the purified library was used for pair-end sequencing on Illumina Hiseq 2000 platform.

### ChIP-seq data analysis

Bowtie2 (v2.3.5) was used to map ChIP-seq raw reads to the GRCm38/mm10 mouse reference genome (13). Then SAMtools v1.9 (14) and bamCoverage program in deeptools (15) were used to remove duplicate reads and generate normalized signals. Subsequently, MACS2 program (v2.2.4) was used to call peaks of ChIP-seq data with the corresponding input data as control (16). To call peaks, the default parameters were adopted except broad peak-calling algorithm was used for H3K36me3 and H3K27me3 ChIP-seq data. RPKM was calculated to quantify each peak. Only peaks with log_10_*P*-value >9 and fold-enrichment >3 were considered in the downstream analyses. HOMER suite was used to find peak associated genes and *de novo* motifs (17). The peaks of different ChIP-seq libraries with at least 1 bp overlaps were defined as overlapping peaks. The union of the peaks in WT and Mst KO ESCs were used to identify differential peaks. The peaks with RPKMs of Mst KO ESCs >2-fold higher or 1.5-fold lower than WT ESCs were identified as Mst KO ESCs upregulated and downregulated peaks respectively. Metaplots and heatmaps were plotted by ngsplot program (v 2.63) (18).

### TF enrichments and data visualization

Segmentations to define the chromatin states in ESCs were adopted from previous report (19). YAP enrichment in each segmentation was calculated using Overlap Enrichment program in ChromHMM (20). The enrichment score was calculated as the ratio of the observed versus expected number of overlaps between YAP loci and each chromatin state respectively.

### TF clustering

Hierarchical clustering of the binding sites of the SE associated factors and other factors was performed according to the pairwise enrichment scores as previously described (21). The pairwise enrichment scores at single nucleotide resolution were calculated as the ratios of the observed overlaps versus the expected overlaps based on the binomial background model. ChIP-seq data of SE associated factors and other listed factors were downloaded from GEO database (Suz12, GSE28325; Tex10, GSE66735; Klf4, GSE90895; P300, GSE90895; Hdac1, GSE90895; Brg1, GSE90895; Essrb, GSE90895; Ezh2, GSE94834; Med1, GSE115340; Med12, GSE115340).

### Super-enhancer analysis

ROSE algorithm (11,22) was adopted to identify the SEs using H3K27ac ChIP-seq data of WT ESCs and Mst KO ESCs. The ROSE identified SEs with H3K27ac signal fold changes between WT and Mst KO ESC greater than 2 fold, these SEs were determined as unique SEs. The ROSE command lines were provided in https://github.com/ZJRen9/ChIP-seq_and_ROSE-superEnhance_pipeline.

### RNA-seq library construction and data processing

Total RNA of WT and Mst KO ESCs and day 4 EBs was harvested using Trizol (Invitrogen). Contaminant DNA was removed by RNase-Free DNase (NEB). RNA-seq libraries were prepared with Dynabeads mRNA Purification Kit (Ambion) and TruSeq Stranded mRNA Library Prep Kit (Illumina). Sequencing was carried out with Illumina HiSeq 2000. Raw paired-end sequencing reads were aligned to mouse GRCm38/mm10 reference genome by HISAT2 (2.1.0) (23). FPKMs (fragments per kilobase of transcript per million mapped reads) were calculated using Cufflinks (v2.1.1) (24). The genes with FPKMs>1, fold changes >2, and *q* values <0.01 were identified as the differentially expressed genes. GO and GSEA analyses were performed using Clusterprofile (25).

### Mesoderm directed differentiation

Mesoderm in vitro induction was performed as previously reported (26). 1.5 × 10^5^ ESCs were seeded per well in ultra-low attachment surface six-well plate (Corning) in modified serum-free differentiation (SFD) medium for 2 days and small EBs were formed. The SFD medium is consisted of IMDM (Gibco, 12440053) and Ham-F12 (Cellgro, 10-080-CVR), supplemented with B27 (Gibco, 12587-010) and N2 supplements (Gibco, 17502-048), Glutamine (Gibco, 25030-081), 10% bovine serum albumin (Invitrogen, P2489), Ascorbic acid (Sigma-Aldrich, A4544), and MTG (Sigma-Aldrich, M6145). To direct mesoderm differentiation, EBs were dissociated into single cells by trypsin and 1.5 × 10^5^ cells were seeded per well in ultra-low attachment surface six-well plate (Corning) in SFD medium containing 5ng/ml Activin A (R&D, 338-AC-010), 5ng/ml VEGF (R&D, 293-VE-010), 0.5 ng/ml BMP4 (Stemgent, 03-0007) for 48 h. To trace the middle stage of cell differentiation, 24 and 48 h samples were collected for analysis.

### Generation of YAP KO ESCs

CRISPR/Cas9 plasmid (pSpCas9(BB)-2A-Puro (PX459) V2.0, Addgene #62988, Cambridge, MA, USA) containing guide RNA which targets YAP first exon was used to knock out YAP in Mst KO ESCs. Guide RNAs were designed using a web-based sgRNA design tool (https://crispr.cos.uni-heidelberg.de/). sgRNA oligos were listed in Supplementary Table S1. PX459-sgRNA-YAP constructs were transfected into Mst KO ESCs by lipofectamine 3000. Twenty-four hours after transfection, ESCs were selected by 1 μg/ml puromycin to eliminate the non-transfected cells. The survival cells were sub-cultured and harvested for western blotting and DNA sequencing to analyze YAP knockout efficiency.

### 3D-Structured Illumination microscopy (3D-SIM) for super-enhancers

To visualize SEs, 3D-SIM experiments were performed. Cells were seeded on a chambered cover glass (Lab-Tek, Cat# 155411) and fixed with 4% PFA after 2 days culture. The samples were then permeabilized by 0.1% Triton and blocked with 3% BSA for 1 h. The samples were next blotted with MED1 antibody (Abcam, Cat#Ab60950) at 1:1000 at 4°C overnight and then blotted with Goat anti-Rabbit secondary antibody conjugated with Alexa Fluor 488 (Invitrogen, Cat#A11008) after wash. Nuclei were counterstained with DAPI (1mg/ml) at 1:2000 dilution for 5 min at RT. ProLong Diamond Antifade Mountant (Invitrogen, Cat#P36961) reagent was added to the sample before super-resolution images were taken using 100× oil-immersion objective of A1R N-SIM N-STORM microscope (Nikon). To obtain optimal images, immersion oil with refractive indices of 1.516 was used at 25°C room temperature. Super-resolution image stacks were captured with a *z*-distance of 0.125 μm with five phases, three angles and 15 raw images per plane. The raw images were reconstructed into one image using NIS Elements software. All SIM images were cropped and processed by NIS Elements software. YAP antibody (Santa cruz, Cat#sc-101199) was used for YAP/MED1 co-immunostaining at 1:50 dilution and Goat anti-mouse Alexa Fluor 647 (Invitrogen, Cat#A21236) was used as a secondary antibody. Co-localization of the two channels was evaluated with Fiji Coloc 2 plugin. Imaris was used to render all the individual z-plane images and Med1 condensates were calibrated with spherical beads with mean short diameter > 0.7 μm.

### Quantitative real-time reverse transcription PCR

Total RNA was isolated from mouse ESCs using Trizol reagent (Invitrogen). 2 ug RNAs were first digested with DNase I (NEB) to remove contaminated DNA and then reverse transcribed using PrimeScript RT reagent Kit (Takara) to synthesize first-strand cDNA. Real-time quantitative PCR was carried out with gene-specific primers using SYBR Premix Ex Taq Kit (Takara) in Quantstudio 12K Flex real-time PCR system (Applied Biosystems). Ct values for target genes were normalized against Gapdh. The relative expression of target genes was further normalized against control sample and determined by 2^−(ΔΔCt)^ method. Three biological repeats and three technical repeats for each sample were tested for each assay. The primers used in RT-qPCR were listed in Supplementary Table S1.

## RESULTS

### The disturbed lineage gene expression during Mst KO ESC differentiation

We have previously demonstrated that Mst KO ESCs preferentially differentiate into neuroectoderm lineage but show defects in differentiation into mesoderm and endoderm lineage cells. To dissect the mechanisms underlying this bias in differentiation of Mst KO ESCs, we used the embryoid body (EB) differentiation system as our model. ESCs can differentiate and form embryoid bodies (EBs) *in vitro* when cultured in the proper condition. EBs recapitulate the lineage differentiation program of embryos in many aspects, and can serve as a powerful *in vitro* model to explore the mechanisms of early lineage cell differentiation (27). Day 4 EBs resemble embryos at the implantation stage, harboring pluripotent epiblast like cells and progenitor like cells for three germ layers (28). Hence, we adopted EB system to explore the function of Mst1 and Mst2 in early cell lineage differentiation.

**Figure 1.**
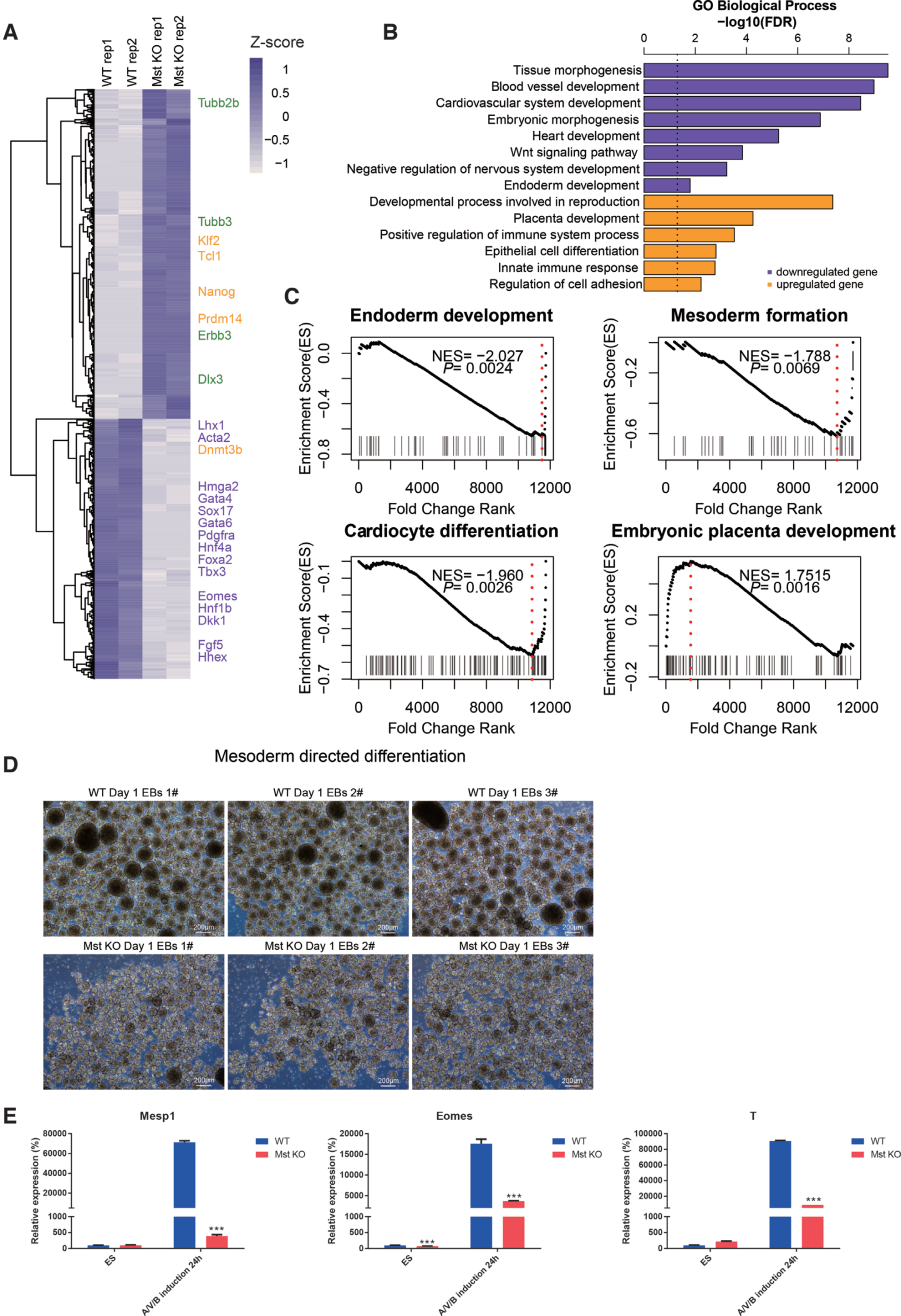
Comparison of gene expression in wild type and Mst KO day 4 EBs and directed mesoderm differentiation. (**A**) Heatmap of RNA-seq expression data of Mst KO and WT day 4 EBs. Representative differentially expressed genes (DEGs) are indicated. (**B**) GO enrichment of upregulated and downregulated genes in day 4 Mst KO versus WT EBs. (**C**) Gene Set Enrichment Analysis (GSEA) of differentially expressed genes between Mst KO and WT day 4 EBs among genes associated endoderm development, mesoderm formation, cardiocyte differentiation and embryonic placenta development. (**D**) Morphology of WT and Mst KO ESC suspension culture after 24 h growth in mesoderm induced medium. Scale = 200 μm. (**E**) Real-time qPCR result of mRNA level of nascent mesodermal genes (*Mesp1*) and mesendoderm marker (*Eomes, T*) in WT and Mst KO samples at ESC stage and 24h after mesoderm directed induction. All data are presented as the mean ± SD (*n* = 3). Gapdh was used as an internal control. Statistically significant differences are indicated (*P< 0.05; **P< 0.01; ***P< 0.001).

By comparing the transcriptome of Mst KO and wild type (WT) day 4 EBs, we found an obvious gene expression alteration with multiple mesoderm and endoderm genes, such as *Hmga2* (29), *Sox17*, *Gata6* and *Gata4*, downregulated in Mst KO EBs (Supplementary Table S2). In the meantime, the critical neuroectoderm markers, such as *Tubb2b*, *Tubb3*, *Errb3* and *Dlx3* were upregulated (30–35). This is consistent with our previous observation that Mst KO ESCs showed impaired mesoderm and endoderm lineage differentiation while preferentially differentiated into neuroectoderm lineage cells (Figure 1A). Gene ontology (GO) analysis of differentially expressed genes between day 4 Mst KO and WT EBs revealed that the downregulated genes were strongly linked to tissue morphogenesis, cardiovascular system development, endoderm development and heart development. Whereas the upregulated genes were linked to placental development, cell adhesion and epithelial cell differentiation (Figure 1B). As expected, gene set enrichment analysis (GSEA) also confirmed that genes related to endoderm development, mesoderm formation and cardiocyte differentiation were downregulated in day 4 Mst KO EBs compared to WT EBs (Figure 1C), while genes associated with embryonic placenta development were inclined to be upregulated in day 4 Mst KO EBs. This is in agreement with the previous report of dramatic increase of trophoblast giant cells in the placenta of Mst KO mouse (36).

To further investigate at which developmental stage Mst1 and Mst2 function, WT and Mst KO ESCs were seeded in mesoderm induction medium which contains Activin A, VEGF and BMP4. It was reported that mesoderm cells start to appear in day 2 ESC formed EBs in this induction medium (26). Just 1 day after induction, Mst KO ESC formed EBs showed obvious smaller size than WT ESC formed EBs (Figure 1D). Early mesoderm marker *Mesp1* was also decreased in Mst KO EBs (Figure 1E). Hence, we deduced that differentiation abnormality might occur at mesendoderm stage, a transient stage before mesoderm formation. It has been reported that Eomes and T are mesendoderm pioneer driving factors that contribute to specify mesendoderm lineage (37). Checking the expression of *Eomes* and *T* by real-time PCR revealed significantly lower expression of these genes in day 1 Mst KO EBs than wild type EBs. These results suggest that Mst1/2 depletion results in differentiation disturbance at as early as mesendoderm stage.

### Co-occupancy of YAP and super-enhancer associated factors in ESCs

**Figure 2.**
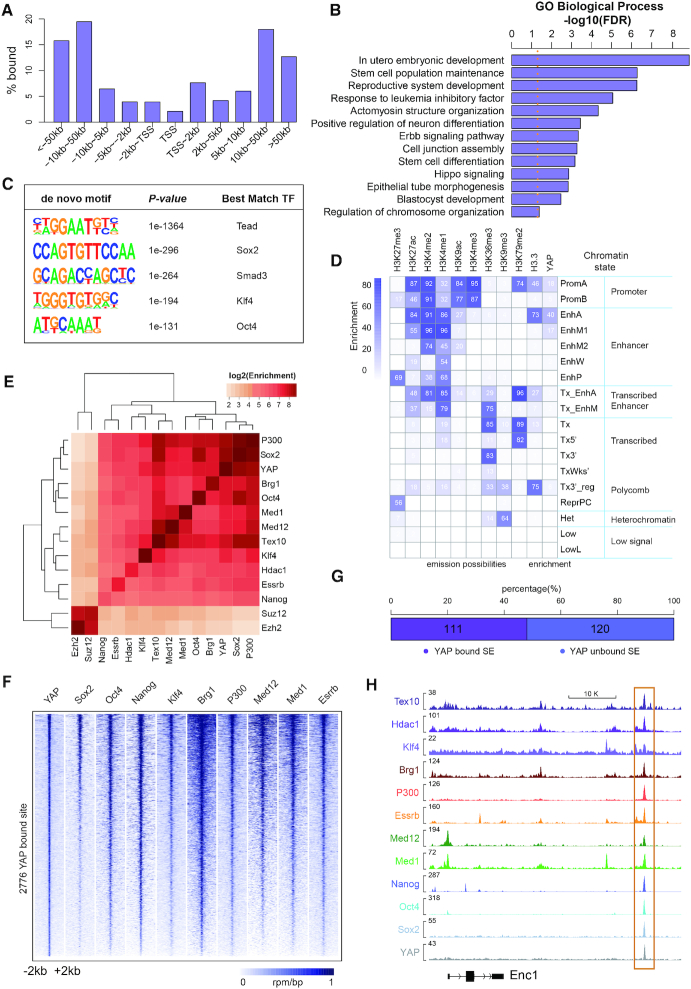
YAP predominantly binds to distal regions from transcription start site (TSS) and shows high correlation with super-enhancer associated factors. (**A**) Distribution of YAP ChIP-seq peaks with respect to the TSSs in WT ESCs. (**B**) GO analysis of genes with YAP enrichment within 50kb of their TSSs in WT ESCs. (**C**) *De novo* motif analysis of YAP peaks. (**D**) YAP enrichment at chromatin sites defined by ChromHMM. Chromatin states and their mnemonics are represented in row. The frequency of indicated histone epigenetic marks and variant at each chromatin state represented as ChromHMM emission probabilities is showed in column. Enrichment is marked from blue (highest) to white (lowest). (**E**) Heatmap demonstrating the enrichment of co-localization between the peaks of YAP and core pluripotency factors, epigenetic modifiers and SE related factors in WT ESCs. Hierarchical clustering dendrograms are shown at the top and left of the heatmap. (**F**) Heatmaps of normalized ChIP-seq signal for Sox2, Oct4, Nanog, Klf4, Brg1, P300, Med12, Med1 and Esrrb at all YAP bound loci in WT ESCs. (**G**) Stacked bar plot showing the proportion of YAP bound and unbound SEs among 231 high-confidence SEs reported in mouse ESCs. (**H**) ChIP-seq signal tracks of YAP and SE related factors at *Enc1* distal enhancer in WT ESCs.

Given the fact that YAP is the major effector of the Hippo signaling and plays an important role in gene expression regulation, we next investigated the role of YAP in ESCs through ChIP-seq assay. We found that YAP were enriched at both gene body and intergenic region, with a strong preference for distal regions away from transcription start sites (TSSs) (Figure 2A, Supplementary Table S3). GO analysis of genes located within ±50 kb from YAP bound sites revealed that YAP tended to bind genes associated with embryonic development, stem cell population maintenance and stem cell differentiation (Figure 2B). YAP also bound to Hippo signaling genes, suggesting a feedback regulatory mechanism of Hippo signaling (Figure 2B). *De novo* motif discovery revealed that binding motifs of Tead family proteins, Sox2, Smad3, Klf4 and Oct4 were enriched in YAP binding loci (Figure 2C). Of note, YAP indirectly binds to DNA with the aid of other transcription factors, such as Tead family proteins, due to lacking of DNA binding domains (38). Enrichment of Tead family protein binding motif in YAP bound sites manifests the reliability of our YAP ChIP-seq result. YAP and Smad3 share the consensus binding motif, also confirming a crosstalk between Hippo signaling and TGF-β signaling reported before (39). In addition, the enrichment of pluripotent factors Sox2, Klf4 and Oct4 binding motif in YAP bound sites suggested that YAP may integrate into the regulatory network of the pluripotent factors. To characterize YAP binding regions, we utilized chromHMM to classify the whole genome into 18 chromatin states according to the histone modifications and H3.3 profiles as previous report (19). Further intersecting YAP bound sites into the chromatin states revealed the most significant enrichment of YAP at acetylated enhancers as well as an obvious enrichment at active promoters and moderately acetylated enhancers (Figure 2D).

As we have found, YAP bound sites were enriched at acetylated enhancers and Sox2, Oct4 and Klf4 binding motif, which usually mark SEs in ESCs (11). Therefore, we hypothesized that YAP might be a SE associated factor. Multiple factors such as Med1, Med12, Tex10, Brg1, Oct4, Sox2, Klf4, P300, Nanog, Essrb and Hdac1 have been reported to be involved in SE formation (11,40,41). Interrogating the binding profiles among YAP and these factors revealed remarkable correlation of YAP with the SE associated factors rather than PRC2 complex subunit Suz12 and Ezh2, which were not related to SEs (Figure 2E). Sox2, Oct4, Nanog, Klf4, Brg1, P300, Med12, Med1 and Esrrb all displayed high co-occupancy at YAP binding loci (Figure 2F). Besides, these SE associated factors showed enhanced enrichment at YAP bound loci than YAP unbound loci (Supplementary Figure S1A). We found that about 48% of previously reported SEs were bound by YAP in WT ESCs (Figure 2G, Supplementary Table S4). These SE regulated genes includes pluripotent genes such as *Smarcad1*, *Esrrb* and *Dppa5a* and neuroectoderm lineage differentiation genes such as *Enc1* and *Brd1* (11,36,42–46) (Figure 2H, Supplementary Figure S1B), indicating that YAP may play a role in lineage differentiation *via* involving in SE modulation in ESCs.

### YAP induces the binding of Nanog/Oct4/Sox2 and H3K27ac modification at YAP bound loci

**Figure 3.**
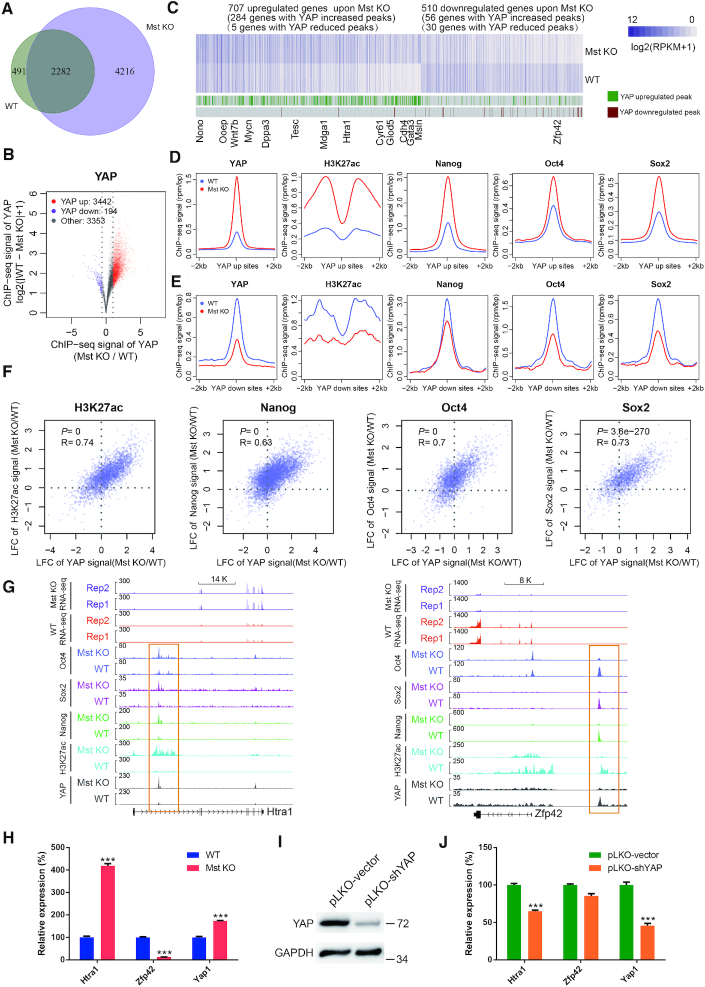
YAP induces the binding of Nanog/Oct4/Sox2 and H3K27ac modification at YAP bound loci. (**A**) Venn diagram showing the overlap of YAP ChIP-seq peaks between WT and Mst KO ESCs. (**B**) Volcano plot showing the differences of YAP ChIP-seq signals between WT and Mst KO ESCs. The plot is based on the union YAP peaks of WT and Mst KO. (**C**) Heatmap of differentially expressed genes between WT and Mst KO ESCs. The genes with upregulated and downregulated YAP binding within 50kb of TSS are indicated below the heatmap. The names of representative genes were labelled at the bottom of the figure. (**D** and **E**) Metaplots of mean ChIP-seq signals of YAP, H3K27ac, Nanog, Oct4 and Sox2 in WT (blue line) and Mst KO (red line) ESCs across the centers and flanking regions of YAP peaks that are upregulated (**D**) or downregulated (**E**) upon Mst1/2 knockout. The plots are based on the union YAP peaks of WT and Mst KO. (**F**) Scatter plots demonstrating the correlations of ChIP-seq signal changes by Mst KO between YAP and H3K27ac, Nanog, Oct4 and Sox2 respectively. The plots are based on the union YAP peaks of WT and Mst KO. LFC: Log2 Fold Change. (**G**) Tracks showing gene expression and the co-localization of peaks of YAP, Nanog, Oct4 and Sox2 as well as H3K27ac modification at *Htra1* and *Zfp42* in WT and Mst KO ESCs. Orange rectangle indicates the bound enhancer region. (**H**) Real-time qPCR showing the expression level of *Htra1*, *Zfp42* and *Yap1* in WT and Mst KO ESCs. Error bars represent standard deviations (n = 3). Gapdh was used as an internal control. Statistically significant differences are indicated (*P< 0.05; **P< 0.01; ***P< 0.001). (**I**) Western blot showing protein level of YAP in YAP knockdown Mst KO ESCs. GAPDH was used as a loading control. (**J**) Real-time qPCR showing the expression level of *Htra1*, *Zfp42* and *Yap1* in control and YAP knockdown Mst KO ESCs. Error bars represent standard deviations (*n* = 3). Gapdh was used as an internal control. Statistically significant differences are indicated (*P< 0.05; **P< 0.01; ***P< 0.001).

To address the potential role of YAP in lineage differentiation regulation, we compared the YAP binding profiles between Mst KO ESCs and WT ESCs. We found that 2282 YAP bound loci were shared by WT and Mst KO ESCs. Other than this, WT ESCs had 491 unique YAP bound loci, while Mst KO ESCs had 4216 unique YAP bound loci. This is in agreement with the increased nuclear accumulation of YAP in Mst KO ESCs (Figure 3A, Supplementary Table S5). Within YAP unique and common bound loci in WT and Mst KO ESCs, YAP preferentially bound the distal regions away from TSSs, particularly in the enhancer regions (Supplementary Figure S2A and B), and showed strong co-localization with SE associated factors (Supplementary Figure S2C). Besides, similar YAP binding motifs were identified within YAP unique and common bound loci in WT and Mst KO ESCs, which were enriched with binding motifs of Tead family, Klf family and Sox family proteins, Oct4 as well as Nr5a2, an Oct4 regulator and a critical regulator of neural stem cell development (47,48) (Supplementary Figure S2D). Of note, the expression level of Tead family genes was not changed between WT and Mst KO ESCs. Among them, Tead1 expressed at the highest level, followed by Tead2 and Tead4, while Tead3 was almost undetectable (Supplementary Figure S2E). The enrichment propensity of Tead proteins at YAP bound loci was not disturbed by Mst depletion either. Tead1 showed highest co-localization with YAP, followed by Tead4 and Tead2 at YAP unique and common bound loci in WT and Mst KO ESCs (Supplementary Figure S2F). This is in line with previous report that Tead family proteins, Tead1 and Tead4 are widely expressed in the inner cell mass of pre-implantation mouse embryos (49).

Further examination of ChIP-seq signals of all YAP bound loci revealed that YAP signal was significantly increased at 3442 loci, while only decreased at 194 loci in Mst KO ESCs as compared to WT ESCs (Figure 3B). GO analysis revealed that Wnt signaling pathway and Ras protein signaling transduction were exclusively enriched in unique YAP bound genes in Mst KO ESCs (Supplementary Figure S2G). These results suggest that Mst KO does not change the binding specificity but the efficiency of YAP. And the binding efficiency change leads to a change of YAP regulated gene population.

As Wnt signaling is one of the main signaling regulating mesoderm differentiation, we investigated whether unique YAP bound Wnt signaling genes in Mst KO ESCs contribute to the defect of mesoderm differentiation. Mst KO led to new YAP peak formation at 36 out of 162 Wnt signaling genes (Supplementary Figure S3A). However, these YAP bound genes showed no obvious expression change in Mst KO EBs compared to WT EBs (Supplementary Figure S3B and C), suggesting that the defect of Mst KO ESC differentiation was not caused by these genes.

We next compared the gene expression changes between Mst KO and WT ESCs. There were 707 upregulated genes and 510 downregulated genes in Mst KO ESCs compared with WT ESCs. Among them, 284 out of 707 upregulated genes were bound by increased YAP peaks upon Mst1/2 knockout. However, only 56 of 510 downregulated genes had YAP enrichment increased upon Mst1/2 knockout (Figure 3C). Cumulative fraction distribution also confirmed that the expression of genes with increased YAP enrichment was significantly higher than the genes with decreased YAP enrichment (*P* = 2.2 × 10^−14^, Wilcoxon test, Supplementary Figure S4A, Table S6). Co-localization of H3K27ac as well as Nanog, Sox2 and Oct4 with upregulated YAP peaks further increased the expression significantly (Supplementary Figure S4B). Of note, the genes with increased YAP binding and upregulated expression in Mst KO ESCs include ectoderm and neural differentiation related genes such as *Msln* (50), *Htra1* (51,52), *Mycn* (53) and *Cdh4* (54). On the other side, *Zfp42*, a gene with reduced YAP binding in Mst KO ESCs showed significantly lower expression in the Mst KO ESCs than WT ESCs (Figure 3C). These results suggest a positive correlation of YAP abundance and gene expression, and this positive correlation may be related to the co-occupancy of YAP with Nanog, Sox2, Oct4 and H3K27ac.

In ESCs, the high intensity of H3K27ac modification is an indicator to discriminate SE from TE (41). In the meantime, multiple master transcription factors, such as Nanog, Oct4, Sox2, Klf4 and Esrrb bind SEs with much higher density than TEs. The co-occupancy of Oct4, Sox2 and Nanog (short for OSN) is another informative feature to define ESC specific SEs (11). Based on the fact that YAP showed strong co-localization with SE associated factors Oct4, Sox2 and Nanog as well as H3K27ac modification in ESCs, we further investigated whether there was any relationship among them. Interestingly, we found a synergistic increase of the average enrichment of H3K27ac, Nanog, Oct4 and Sox2 at YAP bound loci in accordance with the increase of YAP signal in Mst KO compared with WT ESCs and *vice versa* (Figure 3D and E). Even more, there were significant positive correlations of the ChIP-seq signal changes between YAP and Nanog, Oct4, Sox2 as well as H3K27ac at their co-binding loci when compared Mst KO with WT ESCs (Figure 3F). We also observed significantly higher Mst KO induced signals of H3K27ac, Nanog, Oct4 and Sox2 at YAP bound sites than YAP unbound sites, while the enrichment signals of these factors at YAP unbound sites did not show concordant change in Mst KO ESCs compared to WT ESCs. (Supplementary Figure S4C and D). For example, upregulation of *Htra1* expression by Mst KO was correlated with the synergistically increased binding of YAP, Nanog, Sox2, Oct4 and H3K27ac at their regulatory elements. While downregulation of *Zfp42* expression in Mst KO ESCs was associated with the synergistically decreased binding of YAP, Nanog, Sox2, Oct4 and H3K27ac at the regulatory loci (Figure 3G and H). The direct regulation of YAP on *Htra1* was further confirmed by knockdown of YAP in Mst KO ESCs and rescued expression of *Htra1* (Figure 3I and J). These data suggest that YAP may selectively induce the enrichment of Nanog, Sox2, Oct4 and H3K27ac to form new SEs and promote the expression of these SE regulated genes in Mst KO ESCs.

### YAP directs the formation of novel super-enhancers in Mst KO ESCs

**Figure 4.**
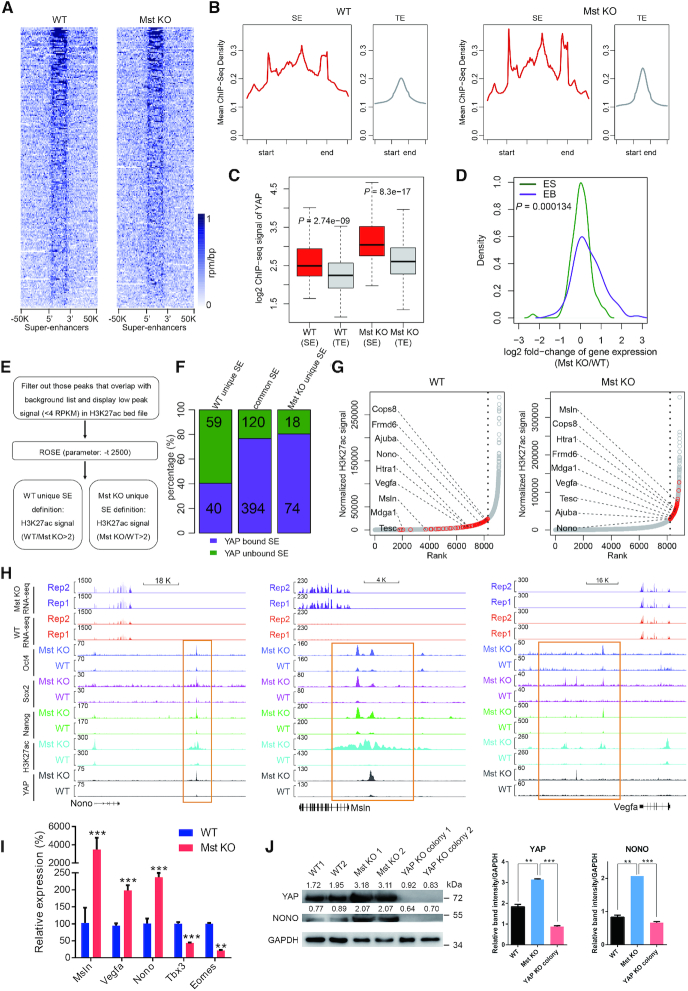
YAP directs the formation of new super-enhancers in Mst KO ESCs. (**A**) The heatmaps of YAP signals at 231 annotated mouse ESC specific SE loci in WT and Mst KO ESCs. (**B**) Metaplots of average YAP ChIP-seq signals at SE and TE loci in WT and Mst KO ESCs. (**C**) Box plot showing YAP signals at SEs and TEs in WT and Mst KO ESCs respectively. (**D**) Density plot comparing the expression change of SE-associated YAP bound genes upon Mst knockout at ES stage (green) and EB stage (purple). (**E**) Illustration of the computational pipeline to identify SEs in WT and Mst KO ESCs using H3K27ac ChIP-seq data. (**F**) Bar-plot showing percentage of YAP bound SEs (purple) and unbound SEs (dark green) that were unique or common in WT and Mst KO ESCs. The exact numbers of YAP bound or unbound SEs are marked within respective colored areas. (**G**) The dot plots showing the distributions of enhancers and SEs sorted and ranked by H3K27ac ChIP-seq signals using ROSE program in WT and Mst KO ESCs respectively. An obvious geometric inflection point was revealed by a dash line. Dots on the left of the dash line represents TEs, while dots on the right of the dash line represents SEs. YAP target genes regulated by TEs in WT ESCs but shifted to be regulated by SEs in Mst KO ESCs are highlighted as red dots. Representative lineage differentiation-associated genes are labelled. (**H**) Track views of ChIP-seq profiles of indicated factors and RNA-seq expression profiles at *Nono*, *Msln* and *Vegfa* in WT and Mst KO ESCs. Newly formed SE upon Mst knockout are marked with orange rectangle. (**I**) Real-time qPCR showing the relative expression level of *Msln*, *Vegfa*, *Nono*, *Tbx3* and *Eomes* in WT and Mst KO ESCs. Error bars represent standard deviations (*n* = 3). Gapdh was used as an internal control. Statistically significant differences are indicated (*P< 0.05; **P< 0.01; ***P< 0.001). (**J**) Western blot showing protein level of YAP and Nono in WT, Mst KO and Mst/YAP double KO ESCs. GAPDH was used as a loading control. The densitometric analyses of YAP and NONO protein levels relative to GAPDH in each sample were shown in western blot assay by ImageJ software.

Global heatmap of YAP binding profile revealed that YAP was enriched at SEs in both wild type and Mst KO ESCs (Figure 4A). Compared to the single and small average YAP peak at TEs, average YAP peak at SEs was broad and high in wild type and Mst KO ESCs (Figure 4B). Mst KO led to increase of YAP signals at both SEs and TEs (Supplementary Figure S5A). However, YAP peak density at SEs was consistently higher than TEs in both WT and Mst KO ESCs (Figure 4C). Among previous reported SEs and TEs, there were significantly higher proportions of YAP bound SEs as compared with TEs in WT ESCs (*P* = 6.70 × 10^−77^, Chi-square test) and Mst KO ESCs (*P* = 1.51 × 10^−59^, Chi-square test) (Supplementary Figure S5B), indicating an important role of YAP in SE regulation. In addition, significantly higher proportions of OSN cobound at YAP bound loci in SEs than YAP bound loci in non-SE regions in both WT (*P* = 3.6 × 10^−7^, Chi-square test) and Mst KO (*P* = 9.9 × 10^−17^, Chi-square test) (Supplementary Figure S5C), suggesting possible synergistic functions of OSN and YAP at SEs in ESCs. Although the function of YAP at TEs is important as well, SEs are more cell-specific than TEs and play key roles in controlling mammalian cell identity (11). We therefore focused on investigating the role of YAP in SE regulation in the following studies.

Tead binding motifs were enriched in YAP bound loci in SEs and different Tead proteins bound to YAP bound loci in SEs at similar ratios at overall YAP bound loci in both WT and Mst KO ESCs (Supplementary Figures S2F and S5D, E). This suggests that Tead family proteins contribute to recruit YAP to their co-bound SE.

Intriguingly, for majority of YAP bound SE regulated genes in mouse ESCs, Mst1 and Mst2 depletion did not obviously increase the gene expression at ES stage. However, global upregulation of these genes appeared in day 4 Mst KO EBs *versus* WT EBs, including genes related to neural lineage differentiation like *Sox2* and *Nr5a2* (47) (Figure 4D, Supplementary Figure S5F). The above data suggested that YAP may function in a similar way as previously reported transcription factors that prime their target genes for subsequent expression change in ESCs (55).

To further test whether YAP involves in remodeling the SEs, we identified and compared the SEs in WT and Mst KO ESCs using ROSE software (56) according to their H3K27ac signals (Figure 4E, F, Supplementary Tables S7 and S8). ROSE identified >70% of previously reported Med1 linked SEs in either WT or Mst KO ESCs (Supplementary Figure S6A), indicating high reliability of ROSE output. GO analysis of genes regulated by YAP bound SEs in WT and Mst KO ESCs revealed that genes associated with positive regulation of neuron differentiation and chromatin organization were specifically enriched in Mst KO ESCs (Supplementary Figure S6B). This is in agreement with the neuroectoderm differentiation propensity observed in Mst KO ESC differentiation.

WT and Mst KO ESCs had 514 common SEs as well as 92 and 99 unique SEs respectively. Of note, YAP bound about 80% of common SEs and Mst KO ESC unique SEs, but only around 40% of WT ESC unique SEs (Figure 4F). In addition, there was no difference of YAP signals between Mst KO ESCs and WT ESCs at WT unique SEs. But for Mst KO unique SEs, YAP signals were significantly higher in Mst KO ESCs than WT ESCs (Supplementary Figure S6C). These results strongly suggest that YAP is linked to new SE formation in Mst KO ESCs.

As anticipated, a subset of genes regulated by TEs in WT ESCs were converted to be regulated by SEs with increased gene expression in Mst KO ESCs (Figure 4G, Supplementary Figure S6D). These genes included *Msln* (50), *Mdga1* (57), *Htra1* (51) and *Tesc* (58), which mark neuroectoderm differentiation; *Nono* (59), *Ajuba* (60) and *Vegfa* (61), which inhibit mesendoderm or downstream lineage differentiation; and YAP negative regulator *Frmd6* (62) (Figure 4G). Mst KO induced significant increase of YAP enrichment on these genes. Concordantly, Oct4, Nanog, Sox2 and H3K27ac enrichment at YAP bound loci was also significantly increased in Mst KO ESCs as compared with WT ESCs. As a result, the expression of these genes was also upregulated in Mst KO ESCs (Figure 4H, Supplementary Figure S6E). These results suggest a remodeling of the SE landscape, converting TEs at key genes to SEs in response to Mst KO.

It is interesting to find that Nono, a Tbx3 repressor (59,63), was regulated by a newly formed YAP bound SE in Mst KO ESCs (Figure 4G and H). Tbx3 directly activates the expression of mesendoderm pioneer factor *Eomes* and plays an pivotal role in mesendoderm specification (63,64). Nono directly binds the promoter of Tbx3 and represses its expression in ESCs (59,63). ChIP-seq assay revealed that Nono but not YAP binds to Tbx3 proximal promoter (Supplementary Figure S7A). Nono expression was significantly upregulated in Mst KO ESCs, accompanying with downregulation of *Tbx3* and *Eomes* (Figure 4I). This result suggests that enhanced YAP activity in Mst KO ESCs promoted the repressive role of Nono on the expression of *Tbx3*, along with downregulation of Tbx3 direct target *Eomes* (64). On the other hand, *Nono* expression level was decreased, while *Tbx3* and *Eomes* level was increased in Mst/YAP double KO ESCs compared to Mst KO ESCs (Supplementary Figure S7B). To further substantiate the regulatory role of Nono on Tbx3, we knocked down *Nono* in Mst KO ESCs (Supplementary Figure S7C). Nono downregulation led to increase of *Tbx3* and *Eomes* without affecting YAP expression (Supplementary Figure S7C). Western blot further confirmed the positive regulation of YAP on Nono at protein level (Figure 4J). Hence, activated YAP in Mst KO ESCs indirectly suppresses the expression of Tbx3 and mesendoderm specification through directing the formation of the novel super-enhancer that activates the expression of Nono. The differentiation distortion of Mst KO ESCs may be partially due to the newly built YAP-Nono-Tbx3 regulatory axis in the absence of Mst1 and Mst2.

In addition, YAP also promoted SE formation at neural progenitor marker *Msln* and cardiac inhibitor gene *Vegfa* and their expression in Mst KO ESCs (Figure 4H and I). As expected, their expression was significantly downregulated in Mst/YAP double KO ESCs compared to Mst KO ESCs (Supplementary Figure S7B), demonstrating that YAP directly regulates the lineage differentiation associated genes *via* newly formed SEs in Mst KO ESCs and accounts for enhanced neural lineage differentiation but impaired cardiac lineage differentiation of Mst KO ESCs to some extent.

To further explore whether YAP was recruited to SE *via* Tead proteins, we knocked down *Tead1*, *Tead2* and *Tead4* individually in Mst KO ESCs by lentivirus expressed shRNAs (Supplementary Figure S7D). Knockdown of *Tead1*, *Tead2* or *Tead4* led to decrease of YAP enrichment at its binding loci in SEs of *Msln*, *Htra1*, *Frmad6* and *Nono* without affecting YAP expression level (Supplementary Figure S7E,F,G), suggesting that Tead1, Tead2 and Tead4 may synergistically recruit YAP to regulate the SEs of the listed genes (Supplementary Figure S7H).

Collectively, these data supported that Mst KO induced upregulation of YAP modulates lineage differentiation *via* establishing novel SEs in Mst KO ESCs.

### YAP promotes Med1 condensates in Mst KO ESCs through phase separation

The phase separation of SE associated factors is mediated by low-complexity disordered regions (LCDRs) or intrinsically disordered regions (IDRs) of these factors. These disordered domains further aggregate together based on hydrophobic interactions with liquid-like properties (65,66). The weak hydrophobic interactions allow for rapid and dynamic regulation of SEs. However, 1,6-hexanediol, which disrupts the hydrophobic interactions, can lead to collapse of this phase separated structure. The mediator complex subunit 1 (Med1) and Brd4 have been found to possess IDR domain that can form liquid-liquid phase-separated condensates at SE elements. Therefore, SEs can be visualized as discrete puncta in cell nuclei by Med1 or Brd4 immuno-fluorescence assay (12).

**Figure 5.**
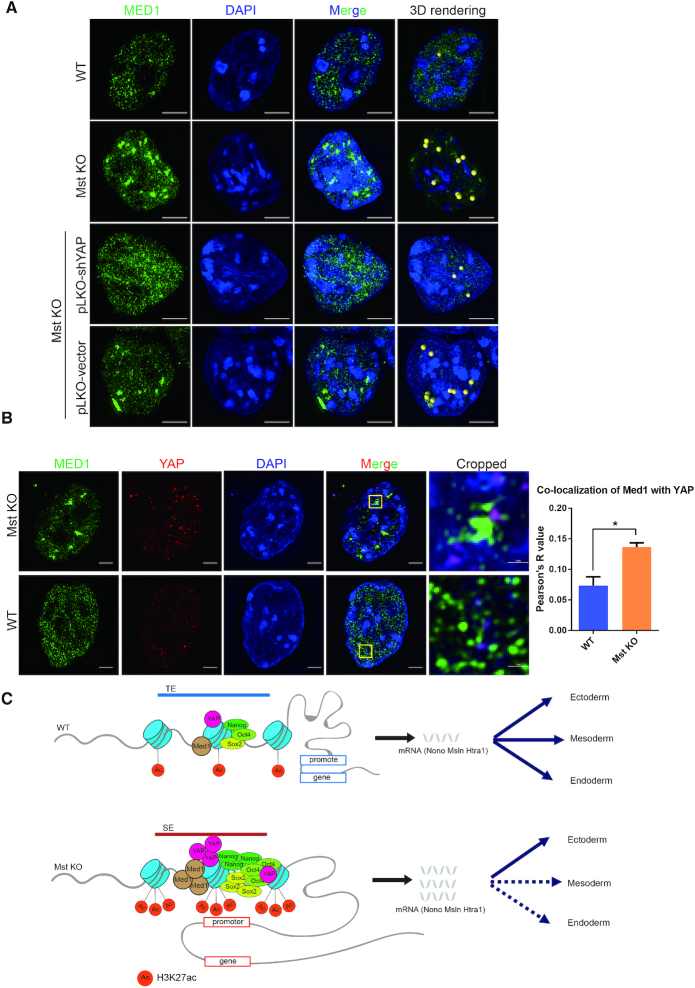
YAP promotes Med1 condensates in Mst KO ESCs through phase separation. (**A**) Immunofluorescence pictures of MED1 puncta in WT and Mst KO ESCs as well as Mst KO ESCs with YAP and control knockdown taken by Nikon super-resolution microscope z-stack mode (3D-SIM). The nuclei were counterstained with DAPI. The rightmost 3D rendering pictures of Med1 phase separated condensates beyond unified criteria were rendered as yellow beads. The scale bars are 5 μm. (**B**) Immunofluorescence pictures of co-immunostaining of WT and Mst KO ESCs with MED1 and YAP antibodies. Pictures were taken by Nikon super-resolution microscope z-stack mode (3D-SIM). The nuclei were counterstained with DAPI. The rightmost cropped pictures show the area in yellow box with magnification. The scale bars are 5 μm and 1 μm in uncropped and cropped pictures respectively. Co-localization of channels for MED1 and YAP was quantified with Fiji Coloc 2 plugin. ROI (region of interest) is chosen in five different nuclei of WT or Mst KO ESCs. Average Pearson's *R* value is used to evaluate co-localization of two channels. (**C**) Model illustrating the mechanism of hyper-activated nuclear YAP in Mst KO ESCs induces preferential lineage differentiation through SEs.

To characterize the role of YAP in SE phase separation, we performed 3D-SIM of Med1 immuno-fluorescence. Significantly larger Med1 puncta could be observed in Mst KO ESCs than WT ESCs. Stable knockdown of YAP by lentivirus expressed shRNA can efficiently rescue Mst KO ESC phenotype and disrupt the super large Med1 puncta. Further quantitation of the large Med1 puncta by performing three-dimensional (3D) rendering and calibration with spherical beads according to unified cut-off criteria such as mean short diameter and mean intensity of puncta revealed about ten Med1 calibrated spheres in Mst KO ESCs and only one sphere in WT ESCs. Knockdown of YAP in Mst KO ESCs significantly reduced Med1 sphere number compared to control knockdown (Figures 3I and 5A). Recently, YAP has been shown to form condensates through phase separation *via* occupying accessible chromatin domains to allow subsequent transcription (67). Co-immunostaining of YAP and Med1 revealed that there were larger YAP puncta in Mst KO ESCs than WT ESCs and YAP puncta showed obvious co-localization with Med1 puncta in Mst KO ESCs (Figure 5B). These results suggest that hyper-activated nuclear YAP may stimulate the change of the 3D SE structure of Med1-labelled condensates.

To confirm that YAP plays a central role in the novel SE formation in Mst KO ESCs, we knocked out YAP in Mst KO ESCs by CRISPR/Cas9 system with two distinct sgRNAs and generated five lines by picking single colonies (Supplementary Figure S8A). Western blot and ChIP assay confirmed the specificity of YAP antibody (Supplementary Figure S8A, B). Next we performed H3K27ac ChIP-seq assay with chromatin from Mst KO ESCs and Mst/YAP double KO ESCs and analyzed SEs with ROSE program. We found that YAP bound more than two third of total SEs in Mst KO ESCs (Supplementary Figure S8C, D). H3K27ac signals at YAP bound SEs were massively downregulated in Mst/YAP double KO ESCs as compared to Mst KO ESCs (Supplementary Figure S8E). The total number of SEs was also significantly decreased in Mst/YAP double KO ESCs (Supplementary Figure S8F). For examples, the peak intensities of H3K27ac at YAP bound SEs of *Msln*, *Htra1* and *Nono* were dramatically reduced in Mst/YAP double KO ESCs as compared to Mst KO ESCs (Supplementary Figure S8G). Hence, YAP deletion significantly diminishes the activity of newly formed YAP bound SEs in Mst KO ESCs.

Taken together, these data suggest that hyper-activation of nuclear YAP by Mst knockout in ESCs results in formation of phase separated novel SEs, leading to a specific and restricted regulation of genes, especially genes that regulate lineage differentiation.

## DISCUSSION

In present study, we explored the molecular mechanism underlying the differentiation distortion of Mst KO ESCs using an *in vitro* mouse ESC to EB differentiation system. As YAP is the effector of Hippo/Mst signaling, we compared the YAP binding profile in genome between WT and Mst KO ESCs and observed an obvious change. This motivated us to explore that relationship between the differentiation distortion of Mst KO ESCs and the dysregulation of YAP. Hyper-active YAP in Mst KO ESCs leads to a global increase of YAP enrichment at genome. About 2 fold of YAP bound sites are created by Mst KO. Enhanced YAP enrichment is generally correlated with increased gene expression, suggesting that YAP mainly plays an active role in gene regulation in ESCs.

Interestingly, YAP tends to bind the enhancer regions of genes. It shows high colocalization with SE associated factors in genome. With increased binding of YAP in Mst KO ESCs, multiple TE regulated genes that promote neuroectoderm differentiation but inhibit mesoderm and endoderm differentiation are switched to be regulated by YAP dependent SEs in Mst KO ESCs. This enables us to propose a Hippo-YAP regulatory model on ESC differentiation. In responding to regular Hippo signaling, YAP which is expressed at a basal level can bind TEs of some lineage specific genes in WT ESCs to balance differentiation of three germ layers. Upon Mst depletion, hyper-activated nuclear YAP triggers synergistical co-localization of high levels of SE associated factors on a subset of neuroectoderm related genes as well as mesendoderm lineage inhibitory genes and disturbs ESC differentiation towards mesoderm and endoderm lineage cells (Figure 5C). 3D-SIM assay also confirmed that YAP promotes Med1 labelled SE condensates in Mst KO ESCs through phase separation. Just like β-catenin of Wnt pathway, STAT3 of JAK/STAT signaling and Smad3 of TGF-β pathway that incorporate into phase-separated MED1 condensates in response to extracellular stimuli (68), YAP may also converge Hippo pathway conveyed growth factor and mechanic stimuli around ESCs into MED1 labelled SE condensates to regulate ESC differentiation.

In this study, we also noticed that YAP binds 80 percent of common SEs shared by WT and Mst KO ESCs. Combined with the fact that Mst KO ESCs can be maintained *in vitro*, it suggests that YAP upregulation in Mst KO ESC doesn’t mess up the major functional machineries in ESCs. It is interesting to observe that multiple pluripotent genes such as *Nanog*, *Dppa5a*, *Sox2* and *Esrrb* regulated by YAP bound SEs, show no difference in expression between WT and Mst KO ESCs, but higher expression in day 4 Mst KO EBs than WT EBs. We found that these pluripotent genes display YAP bound SEs in both WT and Mst KO ESCs, but with increase of YAP enrichment in Mst KO ESCs (Supplementary Table S5). Based on our observation that Mst KO induced change of YAP enrichment triggers a synergistic enrichment change of Nanog, Sox2, Oct4 and H3K27ac in genome (Supplementary Figure S4C), we would expect a relative more potent SE formed in Mst KO ESCs than WT ESCs. But since SE bound pluripotent genes are already expressed at its full engine, it is not surprised that we can hardly detect any expression difference between WT and Mst KO ESCs. However, when ESCs are subjected to differentiation, a more potent SE in Mst KO ESCs would prime its regulated gene to be more resistant to gene silence than WT ESCs. Of course, this is a deduction based on our discovery in this study. Further exploration with experiment would be needed to draw a solid conclusion in the future.

Overall, our study has integrated Hippo-YAP signaling into the key transcriptional regulatory circuitry of ESCs and revealed a novel mechanism on lineage differentiation *via* modulating YAP bound SE formation.

## DATA AVAILABILITY

All raw high-throughput sequencing data have been deposited in the NCBI Gene Expression Omnibus database (https://www.ncbi.nlm.nih.gov/geo/) under the accession number GSE129721.

## Supplementary Material

gkaa482_Supplemental_Files
