# Supplementary material for: Hippo-YAP signaling controls lineage differentiation of mouse embryonic stem cells through modulating the formation of super-enhancers
Source: Nucleic Acids Res. 2020 Jun 8;48(13):7182–96. doi: 10.1093/nar/gkaa482 (PMC7367178; doi:10.1093/nar/gkaa482)
Supplement: gkaa482_Supplemental_Files [file gkaa482_supplemental_files.zip › 2020.5.21 sup.pdf]

Supplementary Figures

Figure S1: YAP binding shows high correlation with super-enhancer associated factors.

(A) Box plots showing log2 enrichment of indicated factors in WT ESCs. The binding sites of these indicated factors are separated into YAP bound and YAP unbound cohorts based on the presence or absence of YAP, respectively. **(B)** Tracks displaying the ChIP-seq signals of YAP, Nanog, Oct4, Sox2 and super-enhancer related factors on *Esrrb*, *Smarcad1*, *Brd1* and *Dppa5a* gene loci in WT ESCs. Orange rectangles indicate the previously identified super-enhancer regions.

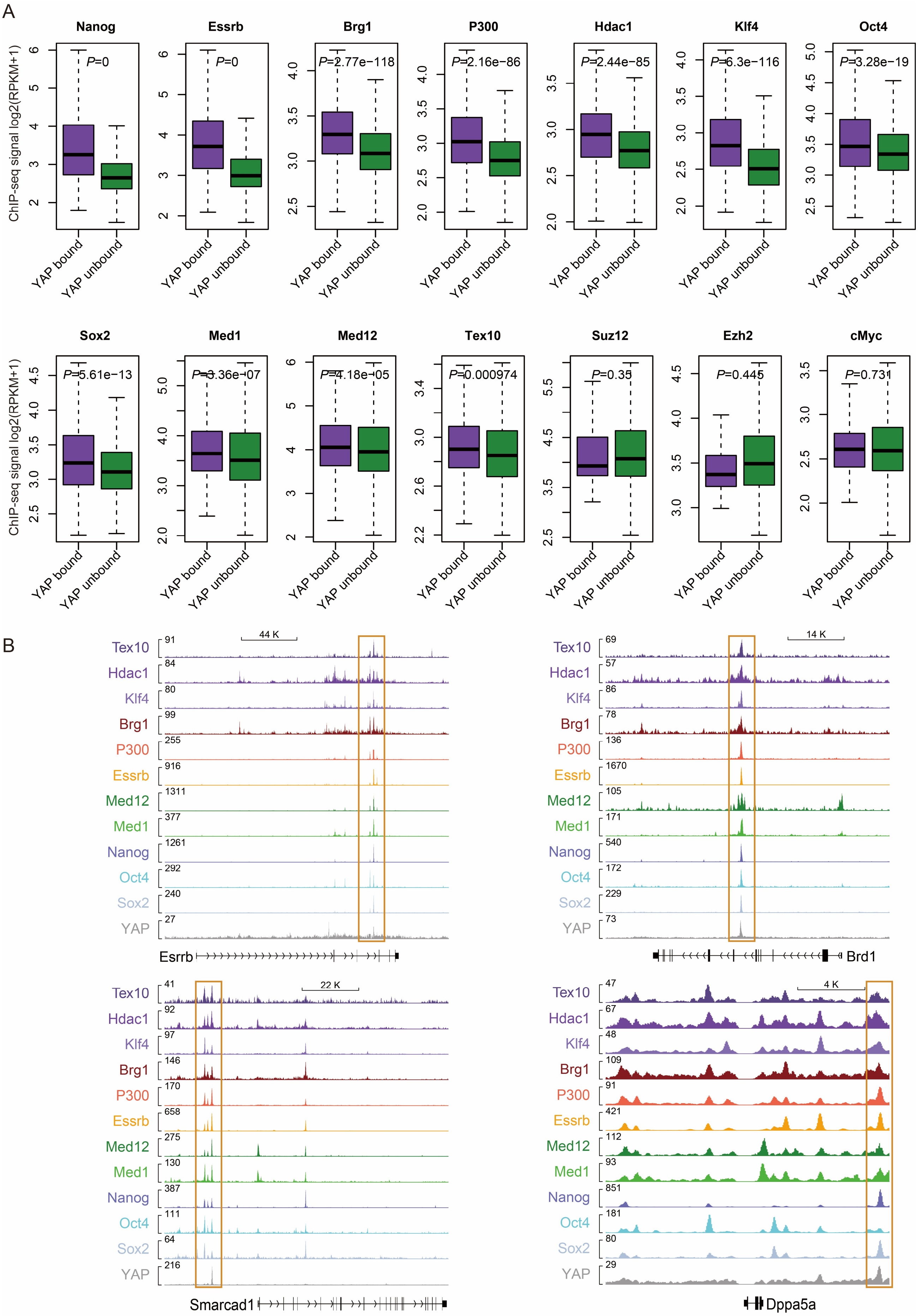
A
Nanog
Essrb
Brg1
P300
Hdac1
Klf4
Oct4
ChIP-seq signal log2(RPKM+1)
P=0
P=0
P=2.77e−118
P=2.16e−86
P=2.44e−85
P=6.3e−116
P=3.28e−19
YAP bound
YAP unbound
Sox2
Med1
Med12
Tex10
Suz12
Ezh2
cMyc
P=5.61e−13
P=3.36e−07
P=4.18e−05
P=0.000974
P=0.35
P=0.445
P=0.731
B
Tex10
Hdac1
Klf4
Brg1
P300
Essrb
Med12
Med1
Nanog
Oct4
Sox2
YAP
44 K
Esrrb
14 K
Brd1
22 K
Smarcad1
4 K
Dppa5a

Figure S2: Propensities of YAP unique and common binding loci in genome in WT and Mst KO ESCs

(A) Distributions of WT and Mst KO unique and common YAP peaks from TSSs. **(B)** YAP enrichment in respect to different chromatin states defined by ChromHMM. Chromatin states and their mnemonics are represented in row. The frequency of indicated histone epigenetic marks and variant at each chromatin state (ChromHMM emission probabilities) is showed in column. Enrichment is marked from blue (highest) to white (lowest). YAP enriched peaks were divided into YAP unique peaks and YAP common peaks in Mst KO and WT ESCs. **(C)** Hierarchical clustering heatmaps of pairwise binding site correlation among YAP, core pluripotency factors Nanog, Oct4 and Sox2, epigenetic modifiers Ezh2, Suz12 and Hdac1 as well as super-enhancer related factors Esrrb, Klf4, P300, Tex10, Med1, Med12 and Brg1. YAP peaks were divided into YAP unique peaks and YAP common peaks in Mst KO and WT ESCs respectively. **(D)** *De novo* motif analysis of YAP consensus binding sites and the matching TF binding motifs. YAP binding sites were divided into YAP unique peaks and YAP common peaks in Mst KO and WT ESCs respectively. **(E)** RT-qPCR measured expression level of Tead family members in WT and Mst KO ESCs. All data are presented as mean ± SD (n=3). Gapdh was used as an internal control. Statistically significant differences are indicated (*, $P<0.05$; **, $P<0.01$; ***, $P<0.001$). **(F)** Bar plots showing the percentage of YAP binding sites that are colocalized with mouse Tead1/2/4 binding sites. YAP binding sites were divided into YAP unique peaks and YAP common peaks in Mst KO and WT ESCs respectively. **(G)** GO analysis of genes with YAP enrichment within 50kb of their TSSs in ESCs. YAP binding sites were divided into YAP unique peaks and YAP common peaks in Mst KO and WT ESCs respectively.

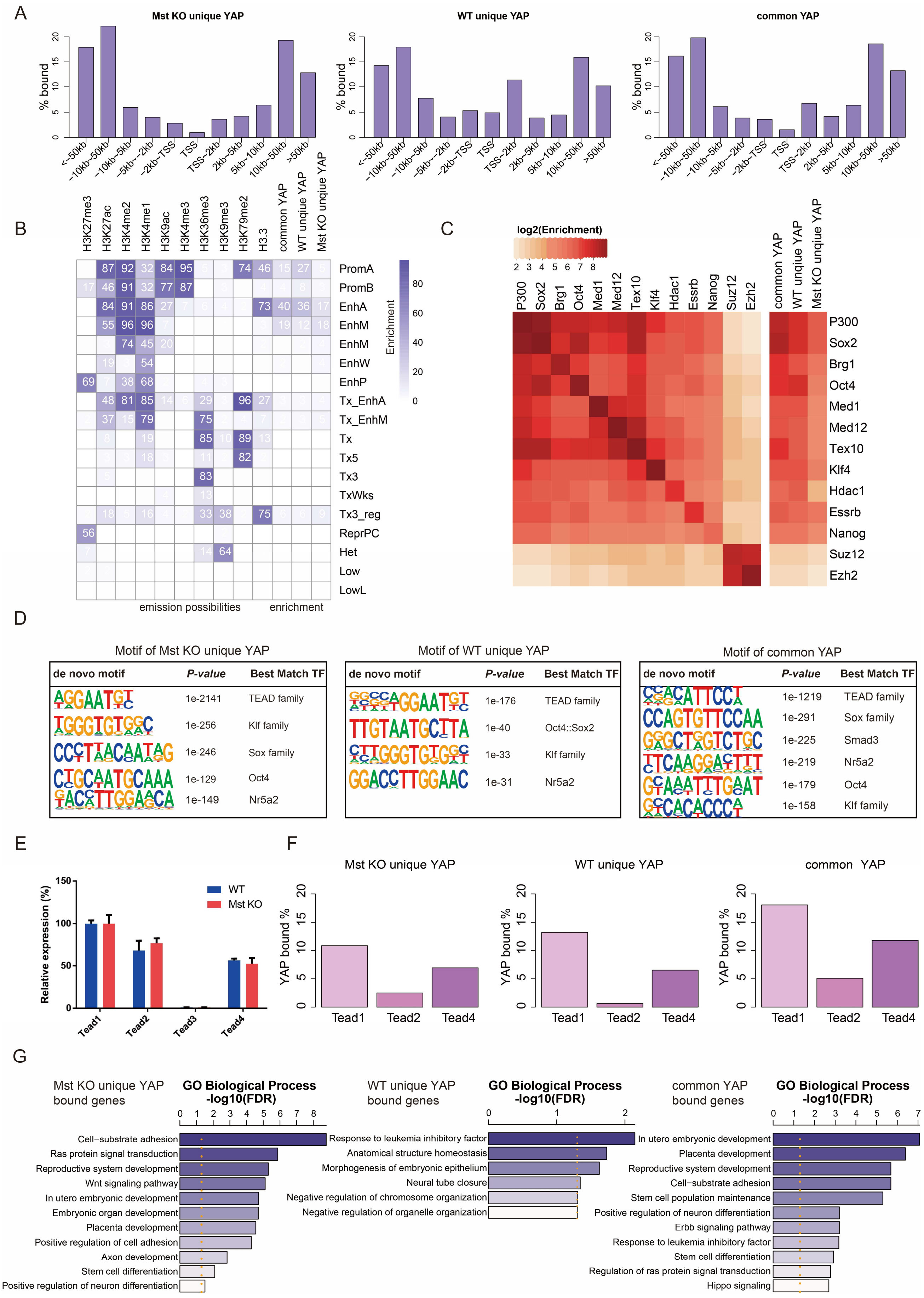
A
Mst KO unique YAP
WT unique YAP
common YAP
% bound
B
emission possibilities
enrichment
Enrichment
C
log2(Enrichment)
D
Motif of Mst KO unique YAP
Motif of WT unique YAP
Motif of common YAP
de novo motif
P-value
Best Match TF
E
Relative expression (%)
WT
Mst KO
F
YAP bound %
G
Mst KO unique YAP bound genes
WT unique YAP bound genes
common YAP bound genes
GO Biological Process -log10(FDR)

Figure S3: Regulatory relationship between Wnt signaling pathway and YAP in Mst KO ESCs.

(A) The pie chart showing the YAP binding profile on Wnt signaling pathway genes download from KEGG website (mmu:04310). Wnt pathway genes with Mst KO unique YAP binding were marked with blue color, of which, several key components of wnt pathway were listed. **(B)** Heatmap showing the expression profile of Wnt pathway genes with Mst KO unique YAP binding between WT EBs and Mst KO EBs. **(C)** Track views showing ChIP-seq enrichment of YAP, H3K27ac, Nanog, Sox2 and Oct4 at Wnt8b and Wnt10a loci bearing newly formed YAP peaks upon Mst knockout (marked by orange rectangle) and RNA-seq profiles between WT and Mst KO EBs.

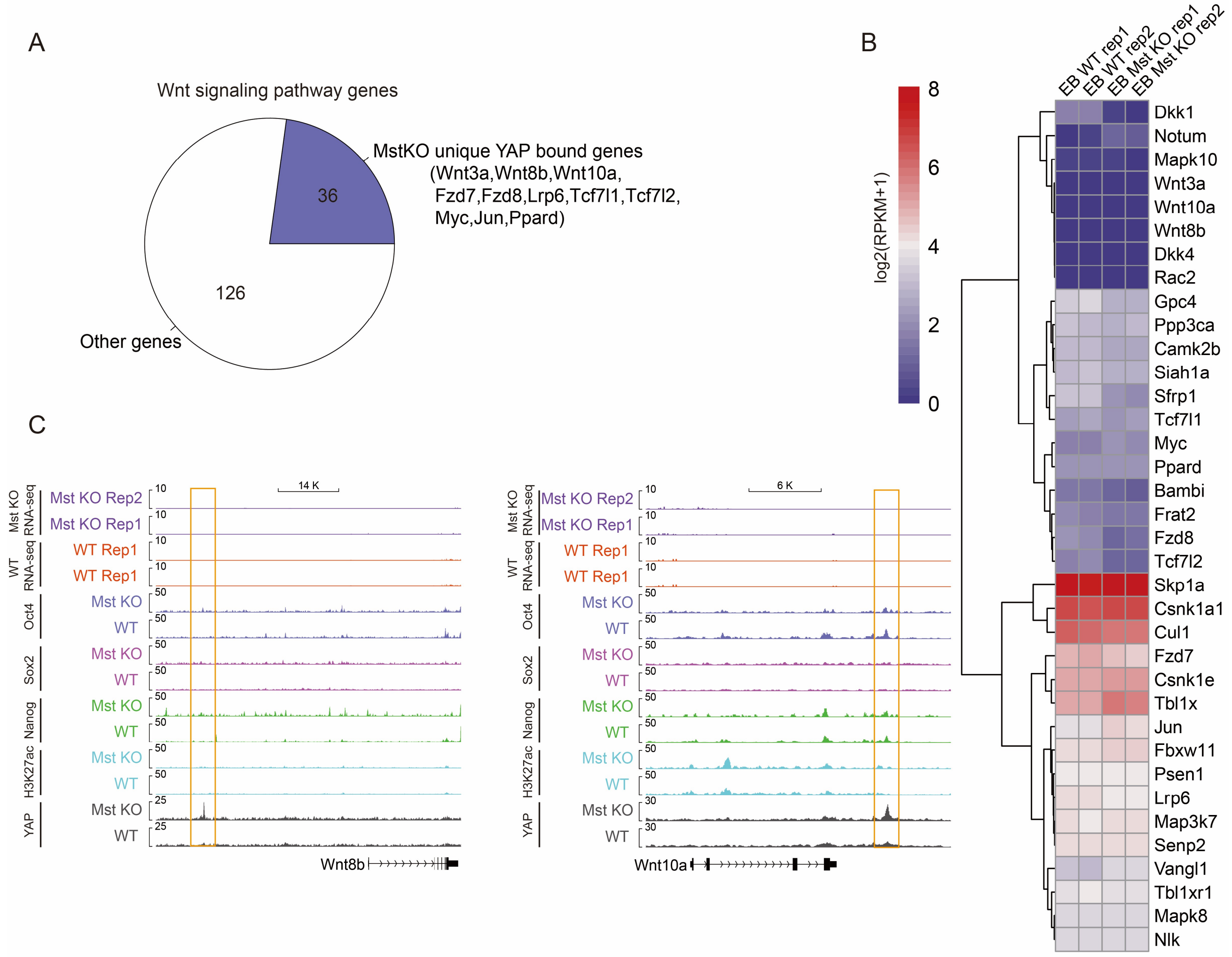
A
Wnt signaling pathway genes
MstKO unique YAP bound genes
(Wnt3a,Wnt8b,Wnt10a,
Fzd7,Fzd8,Lrp6,Tcf7l1,Tcf7l2,
Myc,Jun,Ppard)
36
126
Other genes
B
EB WT rep1
EB WT rep2
EB Mst KO rep1
EB Mst KO rep2
log2(RPKM+1)
8
6
4
2
0
Dkk1
Notum
Mapk10
Wnt3a
Wnt10a
Wnt8b
Dkk4
Rac2
Gpc4
Ppp3ca
Camk2b
Siah1a
Sfrp1
Tcf7l1
Myc
Ppard
Bambi
Frat2
Fzd8
Tcf7l2
Skp1a
Csnk1a1
Cul1
Fzd7
Csnk1e
Tbl1x
Jun
Fbxw11
Psen1
Lrp6
Map3k7
Senp2
Vangl1
Tbl1xr1
Mapk8
Nlk
C
Mst KO RNA-seq
WT RNA-seq
Oct4
Sox2
Nanog
H3K27ac
YAP
Mst KO Rep2
Mst KO Rep1
WT Rep1
WT Rep1
Mst KO
WT
14 K
6 K
Wnt8b
Wnt10a

Figure S4: YAP orchestrates target gene expression program cooperated with H3K27ac modification and Nanog/Oct4/Sox2 core network.

(A) Plot showing the cumulative fractions of the gene expression changes of YAP targeted genes upon Mst knockout. All YAP targeted genes are separated into three catalogs: genes with downregulated, upregulated and unchanged YAP peak upon Mst KO. **(B)** Box plots showing the expression change of YAP targeted genes with upregulated YAP peaks in Mst KO ESCs compared to WT ESCs (short for YAP upregulated). These target genes were further separated into enriched (purple) and not-enriched (red) genes by indicated factors. **(C)** Box plots showing the log2 fold change of ChIP-seq signals for the indicated factors in Mst KO ESCs relative to WT ESCs. Peaks for each factor are separated into YAP bound (purple) and YAP unbound (red). **(D)** Box plots showing ChIP-seq signals of indicated factors at the loci without YAP colocalization (corresponding to the YAP unbound subset in Supplementary Figure S4C) in WT and Mst KO ESCs respectively.

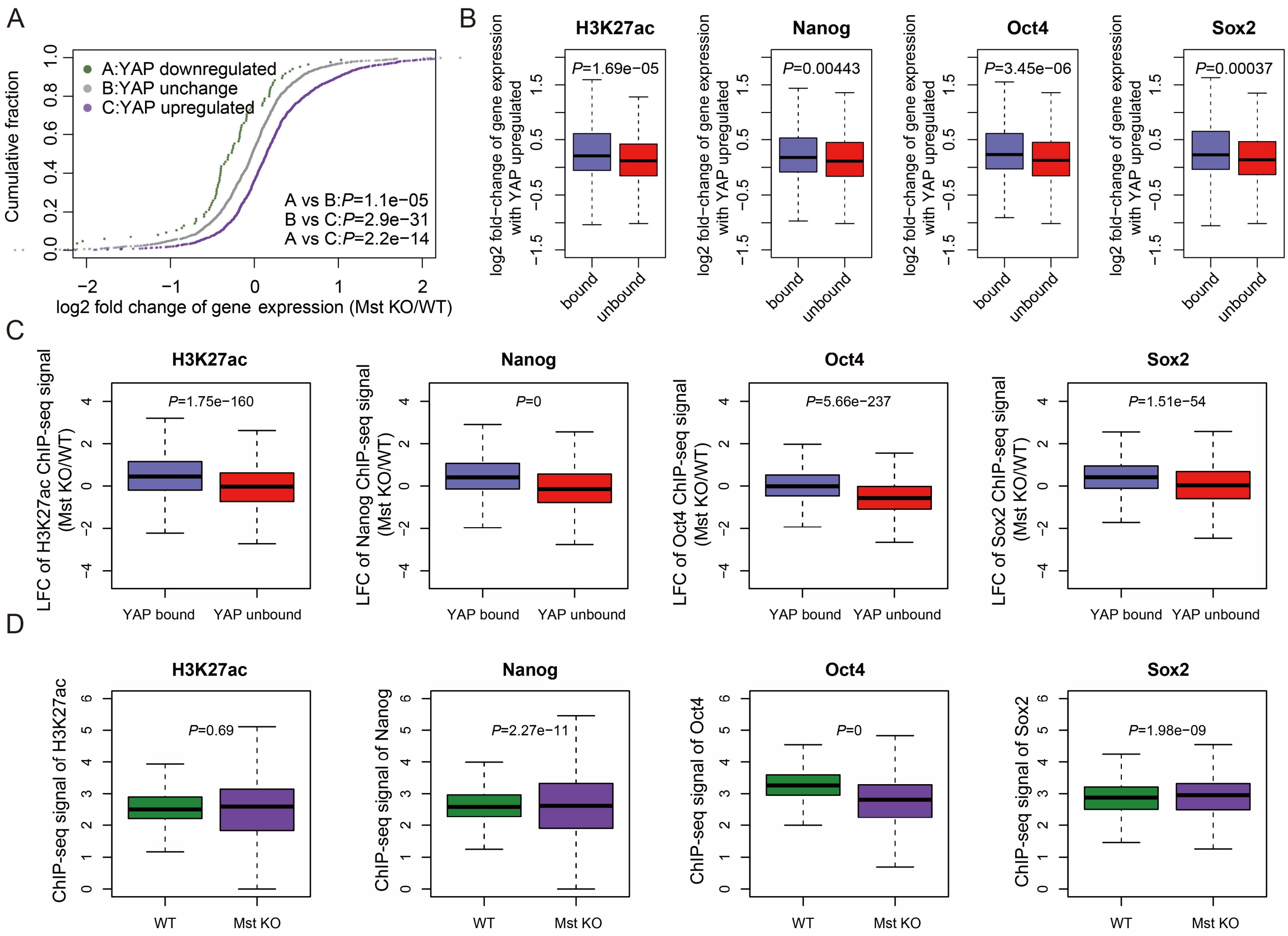

A
Cumulative fraction
A:YAP downregulated
B:YAP unchange
C:YAP upregulated
A vs B:P=1.1e−05
B vs C:P=2.9e−31
A vs C:P=2.2e−14
log2 fold change of gene expression (Mst KO/WT)
B
H3K27ac
Nanog
Oct4
Sox2
P=1.69e−05
P=0.00443
P=3.45e−06
P=0.00037
log2 fold−change of gene expression with YAP upregulated
bound
unbound
C
H3K27ac
Nanog
Oct4
Sox2
P=1.75e−160
P=0
P=5.66e−237
P=1.51e−54
LFC of H3K27ac ChIP-seq signal (Mst KO/WT)
LFC of Nanog ChIP-seq signal (Mst KO/WT)
LFC of Oct4 ChIP-seq signal (Mst KO/WT)
LFC of Sox2 ChIP-seq signal (Mst KO/WT)
YAP bound
YAP unbound
D
H3K27ac
Nanog
Oct4
Sox2
P=0.69
P=2.27e−11
P=0
P=1.98e−09
ChIP-seq signal of H3K27ac
ChIP-seq signal of Nanog
ChIP-seq signal of Oct4
ChIP-seq signal of Sox2
WT
Mst KO

Figure S5: YAP tends to occupy and pre-mark super-enhancers for subsequent upregulation of the expressions of the targeted genes.

(A) Boxplot showing the log2 fold changes of YAP signals in Mst KO versus WT ESCs at YAP bound SEs and TEs respectively. **(B)** Stacked bar plots showing the percentages of YAP bound super-enhancers (SEs) and YAP bound typical enhancers (TEs) in WT and Mst KO ESCs respectively (Purple: YAP bound enhancers; dark green: YAP unbound enhancers). The exact numbers of YAP bound or unbound enhancers are marked within respective colored areas. **(C)** Stacked bar plots showing the proportions of YAP bound SE regions (labelled as SE region YAP) with OSN (Oct4/Sox2/Nanog) co-binding and YAP bound non-SE regions (labelled as Other region YAP) with OSN co-binding in WT and Mst KO respectively. Red: with OSN binding, Blue: without OSN binding. The exact numbers of OSN bound or unbound sites are marked within respective colored areas. **(D)** Bar plots showing the percentage of Tead1/2/4 colocalized at annotated YAP bound SE regions in WT and Mst KO ESCs. **(E)** *De novo* motif analysis of YAP consensus binding sites and the matching TF binding motifs at SEs in WT and Mst KO ESCs. **(F)** Heatmap showing the expression fold change of SE-associated YAP bound genes in Mst KO versus WT in ESCs (first two columns) and day 4 EBs (last two columns).

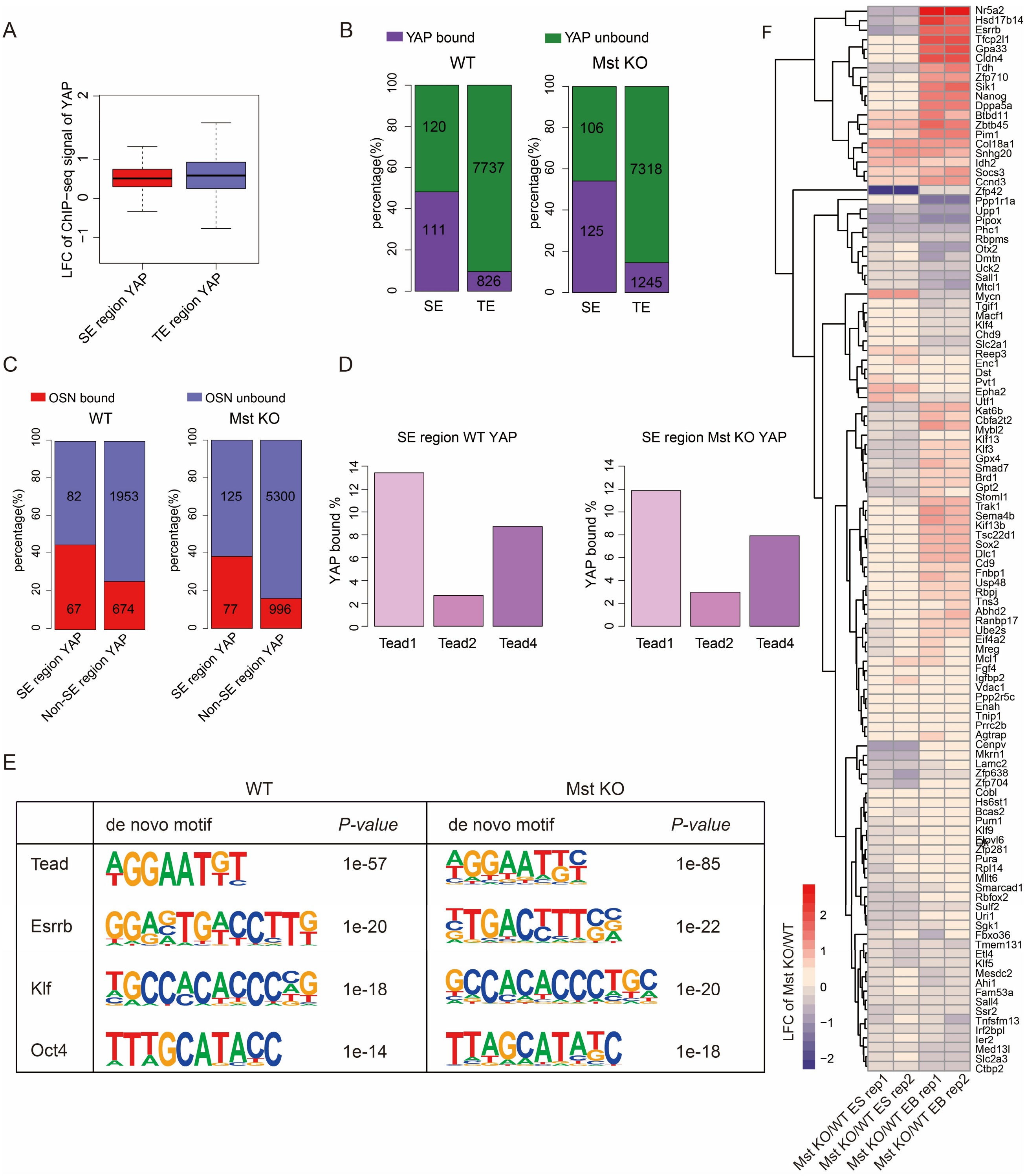
A
LFC of ChIP−seq signal of YAP
−1
0
1
2
SE region YAP
TE region YAP
B
YAP bound
YAP unbound
WT
Mst KO
percentage(%)
0
20
40
60
80
100
120
111
7737
826
106
125
7318
1245
SE
TE
C
OSN bound
OSN unbound
WT
Mst KO
percentage(%)
82
67
1953
674
125
77
5300
996
SE region YAP
Non-SE region YAP
D
SE region WT YAP
SE region Mst KO YAP
YAP bound %
0
2
4
6
8
10
12
14
Tead1
Tead2
Tead4
E
WT
Mst KO
de novo motif
P-value
Tead
1e-57
1e-85
Esrrb
1e-20
1e-22
Klf
1e-18
1e-20
Oct4
1e-14
1e-18
F
LFC of Mst KO/WT
2
1
0
−1
−2
Mst KO/WT ES rep1
Mst KO/WT ES rep2
Mst KO/WT EB rep1
Mst KO/WT EB rep2
Nr5a2
Hsd17b14
Esrrb
Tfcp2l1
Gpa33
Cldn4
Tdh
Zfp710
Sik1
Nanog
Dppa5a
Btbd11
Zbtb45
Pim1
Col18a1
Snhg20
Idh2
Socs3
Ccnd3
Zfp42
Ppp1r1a
Upp1
Pipox
Phc1
Rbpms
Otx2
Dmtn
Uck2
Sall1
Mtcl1
Mycn
Tgif1
Macf1
Klf4
Chd9
Slc2a1
Reep3
Enc1
Dst
Pvt1
Epha2
Utf1
Kat6b
Cbfa2t2
Mybl2
Klf13
Klf3
Gpx4
Smad7
Brd1
Gpt2
Stoml1
Trak1
Sema4b
Kif13b
Tsc22d1
Sox2
Dlc1
Cd9
Fnbp1
Usp48
Rbpj
Tns3
Abhd2
Ranbp17
Ube2s
Eif4a2
Mreg
Mcl1
Fgf4
Igfbp2
Vdac1
Ppp2r5c
Enah
Tnip1
Prrc2b
Agtrap
Cenpv
Mkrn1
Lamc2
Zfp638
Zfp704
Cobl
Hs6st1
Bcas2
Pum1
Klf9
Elovl6
Zfp281
Pura
Rpl14
Mllt6
Smarcad1
Rbfox2
Sulf2
Uri1
Sgk1
Fbxo36
Tmem131
Etl4
Klf5
Mesdc2
Ahi1
Fam53a
Sall4
Ssr2
Tnfsfm13
Irf2bpl
Ier2
Med13l
Slc2a3
Ctbp2

Figure S6: Activation of YAP leads to the formation of new super-enhancers in Mst KO ESCs.

(A) Venn diagrams showing the overlap of previously reported super-enhancers identified by Med1 ChIP-seq in mouse ESCs and super-enhancers identified by ROSE using H3K27ac ChIP-seq in WT and Mst KO mouse ESCs respectively. **(B)** GO analysis of YAP bound SE-associated genes in WT and Mst KO ESCs respectively. **(C)** Box plots comparing YAP signals in WT and Mst KO at WT ESC unique SEs (left) and Mst KO ESC unique SEs (right) respectively. **(D)** Heatmap showing the expression level of genes associated with newly formed YAP bound SEs upon Mst knockout. **(E)** Top panel: ChIP-seq track views of indicated SE associated factors and YAP at *Mdge1*, *Frmd6* and *Tesc* in WT ESCs (marked by orange rectangle). Bottom panel: track views showing ChIP-seq enrichment of YAP, H3K27ac, Nanog, Sox2 and Oct4 at *Mdge1*, *Frmd6* and *Tesc* that have new SE formed upon Mst knockout (marked by orange rectangle) and RNA-seq profiles in WT and Mst KO ESCs.

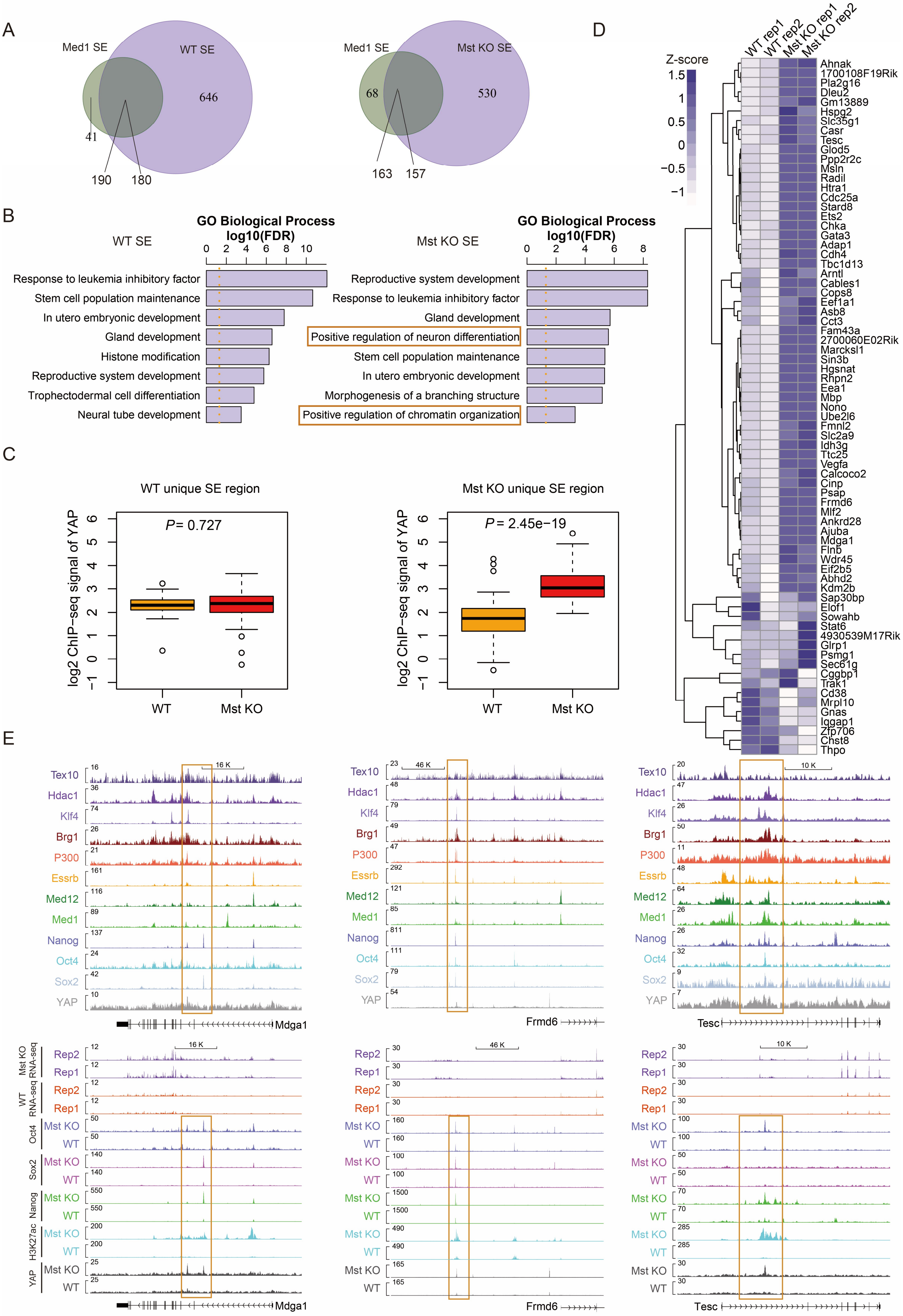
A
Med1 SE
WT SE
646
41
190
180
Med1 SE
Mst KO SE
68
530
163
157
B
WT SE
GO Biological Process
log10(FDR)
0 2 4 6 8 10
Response to leukemia inhibitory factor
Stem cell population maintenance
In utero embryonic development
Gland development
Histone modification
Reproductive system development
Trophectodermal cell differentiation
Neural tube development
Mst KO SE
GO Biological Process
log10(FDR)
0 2 4 6 8
Reproductive system development
Response to leukemia inhibitory factor
Gland development
Positive regulation of neuron differentiation
Stem cell population maintenance
In utero embryonic development
Morphogenesis of a branching structure
Positive regulation of chromatin organization
C
WT unique SE region
P= 0.727
log2 ChIP−seq signal of YAP
WT
Mst KO
Mst KO unique SE region
P= 2.45e−19
WT
Mst KO
D
WT rep1
WT rep2
Mst KO rep1
Mst KO rep2
Z-score
1.5
1
0.5
0
−0.5
−1
Ahnak
1700108F19Rik
Pla2g16
Dleu2
Gm13889
Hspg2
Slc35g1
Casr
Tesc
Glod5
Ppp2r2c
Msln
Radil
Htra1
Cdc25a
Stard8
Ets2
Chka
Gata3
Adap1
Cdh4
Tbc1d13
Arntl
Cables1
Cops8
Eef1a1
Asb8
Cct3
Fam43a
2700060E02Rik
Marcksl1
Sin3b
Hgsnat
Rhpn2
Eea1
Mbp
Nono
Ube2l6
Fmnl2
Slc2a9
Idh3g
Ttc25
Vegfa
Calcoco2
Cinp
Psap
Frmd6
Mlf2
Ankrd28
Ajuba
Mdga1
Flnb
Wdr45
Eif2b5
Abhd2
Kdm2b
Sap30bp
Elof1
Sowahb
Stat6
4930539M17Rik
Glrp1
Psmg1
Sec61g
Cggbp1
Trak1
Cd38
Mrpl10
Gnas
Iqgap1
Zfp706
Chst8
Thpo
E
Tex10
Hdac1
Klf4
Brg1
P300
Essrb
Med12
Med1
Nanog
Oct4
Sox2
YAP
16 K
Mdga1
46 K
Frmd6
10 K
Tesc
Mst KO RNA-seq
WT RNA-seq
Rep2
Rep1
Oct4
Sox2
Nanog
H3K27ac
YAP
Mst KO
WT

Figure S7: YAP enrichment changes upon Tead1/2/4 knockdown in Mst KO ESCs.

(A) Track view showing YAP binding, H3K27ac and H3K27me3 modification as well as RNA-seq at Tbx3 gene locus in WT and Mst KO ESCs. Track of Nono enrichment at Tbx3 was adopted from published data (GSE73426). **(B)** Real time-qPCR showing the expression level of *Msln*, *Vegfa*, *Nono, Tbx3* and *Eomes, Ctgf* in Mst/YAP double knockout ESCs. All data are presented as mean ± SD (n=3). *Gapdh* was used as an internal control. Statistically significant differences are indicated (*, P<0.05; **, P<0.01; ***, P<0.001). **(C)** Real-time qPCR result of mRNA level of *Nono*, *Tbx3* and mesendoderm marker (*Eomes*), *Yap1* in Mst KO ESCs with two distinct shRNAs targeting mouse *Nono*, using Mst KO ESCs harboring Scramble shRNA (shNC) as control. All data are presented as the mean ± SD (n=3). Actin was used as an internal control. Statistically significant differences are indicated (*, P<0.05; **, P<0.01; ***, P<0.001). Western blot showing protein level of NONO and YAP in Mst KO ESCs with Nono knockdown. The densitometric analyses of YAP and NONO protein levels relative to GAPDH in each sample were shown in western blot assay by Image J software. **(D)** Real-time qPCR result showing the mRNA level of Tead1, Tead2, Tead4 upon stable knockdown by lentivirus expressed corresponding shRNAs. **(E)** Real-time qPCR result showing the mRNA level of *Yap* upon Tead1, Tead2 and Tead4 knockdown in Mst KO ESCs. All data are presented as mean ± SD (n=3). Actin was used as an internal control. Statistically significant differences are indicated (*, P<0.05; **, P<0.01; ***, P<0.001). **(F)** Western blot showing that YAP protein level upon Tead1, Tead2, Tead4 knockdown in Mst KO ESCs. GAPDH was used as an internal control. The densitometric analyses of YAP protein relative to GAPDH protein were done by using Image J software. **(G)** ChIP-qPCR result showing the enrichment fold change of YAP at YAP bound SE regions upon Tead1/2/4 knockdown in Mst KO ESCs. YAP binding locus at its well-known target gene *Ctgf* was used as the positive control and *Myod1* locus without YAP binding was used as the negative control. Raw Ct values of YAP ChIP DNA were first normalized to Ct value of matching input DNA. Data were then presented as fold enrichment over that of YAP unbound negative control site with two biological replicates and three technical replicates. Statistically significant differences are indicated (*, P<0.05; **, P<0.01; ***, P<0.001). **(H)** Track views showing YAP and Tead1/2/4 binding profile at SE regions of *Msln*, *Nono*, *Htra1* and promoter region of *Ctgf*.

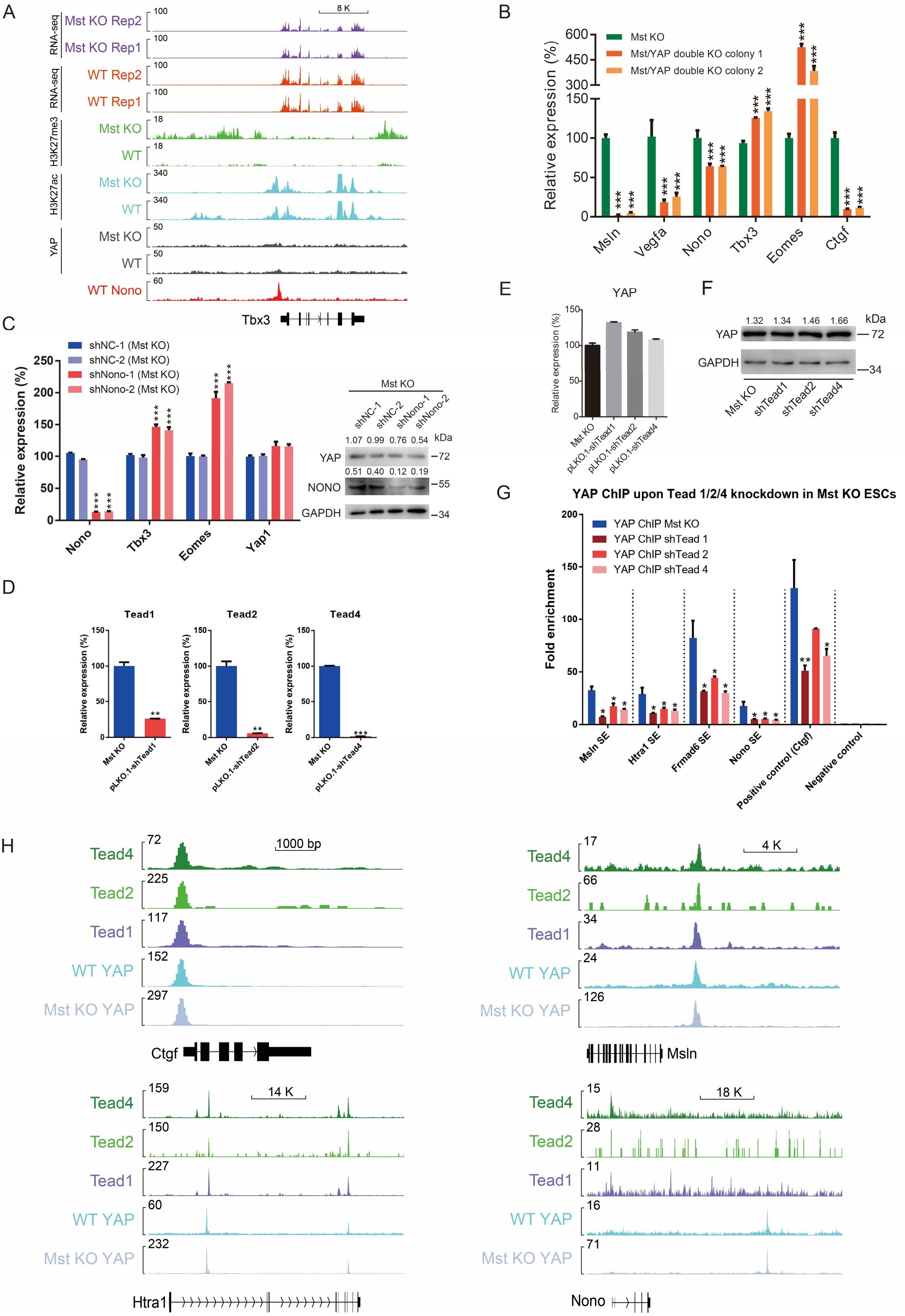

A
8 K
RNA-seq
Mst KO Rep2
Mst KO Rep1
RNA-seq
WT Rep2
WT Rep1
H3K27me3
Mst KO
WT
H3K27ac
Mst KO
WT
YAP
Mst KO
WT
WT Nono
Tbx3
B
Relative expression (%)
Mst KO
Mst/YAP double KO colony 1
Mst/YAP double KO colony 2
Msln
Vegfa
Nono
Tbx3
Eomes
Ctgf
C
Relative expression (%)
shNC-1 (Mst KO)
shNC-2 (Mst KO)
shNono-1 (Mst KO)
shNono-2 (Mst KO)
Nono
Tbx3
Eomes
Yap1
Mst KO
shNC-1
shNC-2
shNono-1
shNono-2
1.07 0.99 0.76 0.54
kDa
YAP
0.51 0.40 0.12 0.19
NONO
GAPDH
D
Tead1
Tead2
Tead4
Relative expression (%)
Mst KO
pLKO.1-shTead1
pLKO.1-shTead2
pLKO.1-shTead4
E
YAP
Relative expression (%)
Mst KO
pLKO.1-shTead1
pLKO.1-shTead2
pLKO.1-shTead4
F
1.32 1.34 1.46 1.66
kDa
YAP
GAPDH
Mst KO
shTead1
shTead2
shTead4
G
YAP ChIP upon Tead 1/2/4 knockdown in Mst KO ESCs
Fold enrichment
YAP ChIP Mst KO
YAP ChIP shTead 1
YAP ChIP shTead 2
YAP ChIP shTead 4
Msln SE
Htra1 SE
Frmad6 SE
Nono SE
Positive control (Ctgf)
Negative control
H
1000 bp
Tead4
Tead2
Tead1
WT YAP
Mst KO YAP
Ctgf
4 K
Msln
14 K
Htra1
18 K
Nono

Figure S8: YAP knockout in Mst KO ESCs dramatically compromises H3K27ac enrichment at SE regions and attenuates the power of SE identification.

(A) Western blot checking YAP protein level in Mst KO ESCs and Mst/YAP double KO ESCs derived from distinct single colonies by transfecting two different sgRNAs. YAP antibodies from Santa Cruz and Novus (for ChIP assay) were employed to detect YAP. GAPDH was used as an internal control. **(B)** ChIP-qPCR result showing the enrichment fold change of YAP at YAP bound Msln SE region and Ctgf promoter region in YAP/Mst double KO ESCs. *Myod1* locus without YAP binding was used as the negative control. Raw Ct values of YAP ChIP DNA were first normalized to Ct value of input DNA. Data were then presented as fold enrichment over that of negative control site with two biological replicates and three technical replicates. Statistically significant differences are indicated (*, $P<0.05$; **, $P<0.01$; ***, $P<0.001$). **(C)** Venn diagram showing the overlap of super-enhancers identified by ROSE according to replicate data of H3K27ac ChIP-seq in Mst KO ESCs. **(D)** Stacked bar plot showing the proportion of YAP bound (purple) and unbound (dark green) SEs among total SEs in Mst KO ESCs according to replicate H3K27ac ChIP-seq data. The exact numbers of YAP bound or unbound SEs are marked within respective colored areas. **(E)** Heatmap showing the H3K27ac intensity at YAP bound SE regions in Mst KO ESCs and Mst/YAP double KO ESCs. **(F)** The dot plot showing distribution of enhancers and super-enhancers sorted and ranked by H3K27ac signals using ROSE program in Mst KO ESCs and Mst/YAP double KO ESCs respectively. An obvious geometric inflection point was revealed by vertical dash line. Dots on the left of the dash line represents typical enhancers, while dots on the right of the dash line represents super-enhancers. Super-enhancers were highlighted as red dots. The number of super-enhancers was labelled. **(G)** Track views of YAP and H3K27ac enrichment at SE regions (marked by orange rectangles) of *Msln*, *Htra1* and *Nono* in Mst KO ESCs and Mst/YAP double KO ESCs. YAP knockout in Mst KO ESCs led to remarkable attenuation of H3K27ac enrichment.

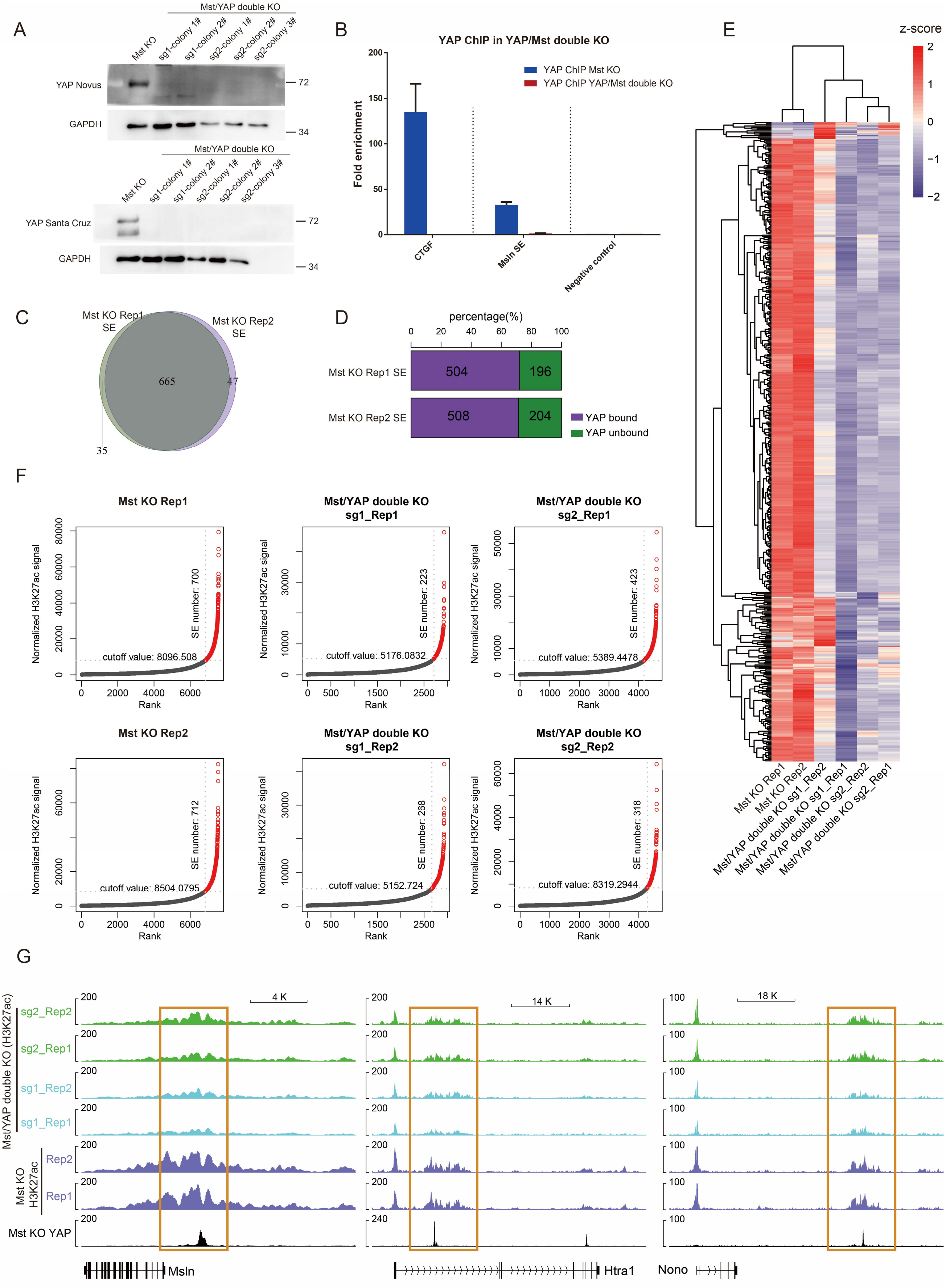
A
Mst/YAP double KO
Mst KO
sg1-colony 1#
sg1-colony 2#
sg2-colony 1#
sg2-colony 2#
sg2-colony 3#
YAP Novus
GAPDH
YAP Santa Cruz
72
34
B
YAP ChIP in YAP/Mst double KO
Fold enrichment
YAP ChIP Mst KO
YAP ChIP YAP/Mst double KO
CTGF
Msln SE
Negative control
C
Mst KO Rep1 SE
Mst KO Rep2 SE
665
47
35
D
percentage(%)
Mst KO Rep1 SE
Mst KO Rep2 SE
504
196
508
204
YAP bound
YAP unbound
E
z-score
Mst KO Rep1
Mst KO Rep2
Mst/YAP double KO sg1_Rep2
Mst/YAP double KO sg1_Rep1
Mst/YAP double KO sg2_Rep2
Mst/YAP double KO sg2_Rep1
F
Mst KO Rep1
Mst/YAP double KO sg1_Rep1
Mst/YAP double KO sg2_Rep1
Mst KO Rep2
Mst/YAP double KO sg1_Rep2
Mst/YAP double KO sg2_Rep2
Normalized H3K27ac signal
Rank
SE number: 700
cutoff value: 8096.508
SE number: 223
cutoff value: 5176.0832
SE number: 423
cutoff value: 5389.4478
SE number: 712
cutoff value: 8504.0795
SE number: 268
cutoff value: 5152.724
SE number: 318
cutoff value: 8319.2944
G
Mst/YAP double KO (H3K27ac)
sg2_Rep2
sg2_Rep1
sg1_Rep2
sg1_Rep1
Mst KO H3K27ac
Rep2
Rep1
Mst KO YAP
4 K
14 K
18 K
Msln
Htra1
Nono
